# Novel 5-Oxopyrrolidine-3-carbohydrazides as Potent Protein Kinase Inhibitors: Synthesis, Anticancer Evaluation, and Molecular Modeling

**DOI:** 10.3390/ijms26073162

**Published:** 2025-03-29

**Authors:** Ingrida Tumosienė, Maryna Stasevych, Viktor Zvarych, Ilona Jonuškienė, Kristina Kantminienė, Vilma Petrikaitė

**Affiliations:** 1 Department of Organic Chemistry, Kaunas University of Technology, Radvilėnų Pl. 19, 50254 Kaunas, Lithuania; ingrida.tumosiene@ktu.lt (I.T.); ilona.jonuskiene@ktu.lt (I.J.); 2 Department of Technology of Biologically Active Substances, Pharmacy, and Biotechnology, Lviv Polytechnic National University, S. Bandera Str. 12, 79013 Lviv, Ukraine; maryna.v.stasevych@gmail.com; 3 Department of Automated Control Systems, Lviv Polytechnic National University, S. Bandera Str. 12, 79013 Lviv, Ukraine; viktor.i.zvarych@lpnu.ua; 4 Department of Physical and Inorganic Chemistry, Kaunas University of Technology, Radvilėnų pl. 19, 50254 Kaunas, Lithuania; 5 Laboratory of Drug Targets Histopathology, Institute of Cardiology, Lithuanian University of Health Sciences, Sukilėlių Pr. 13, 50162 Kaunas, Lithuania; 6 Institute of Biotechnology, Life Sciences Center, Vilnius University, Saulėtekio Al. 7, 10257 Vilnius, Lithuania

**Keywords:** diphenylamine, hydrazone, melanoma, triple-negative breast cancer, pancreatic tumor, 3D cell cultures, migration

## Abstract

A series of novel hydrazones bearing diphenylamine and 5-oxopyrrolidine moieties, along with benzene and naphthalene rings substituted with hydroxy, alkoxy, or carboxylic groups, were synthesized. Their anticancer activity was evaluated in vitro using both 2D (MTT and ‘wound healing’ assays) and 3D (cell spheroid) models against human melanoma IGR39 cells, the triple-negative breast cancer cell line MDA-MB-231, and pancreatic carcinoma Panc-1 cell line. Compounds **8** (2-hydroxybenzylidene derivative) and **12** (2-hydroxynaphthalenylmethylene derivative) demonstrated the highest cytotoxicity in both 2D and 3D assays, while compounds **4** (2,5-dimethoxybenzylidene derivative) and **6** (2,4,6-trimethoxybenzylidene derivative) were most effective at inhibiting cell migration. Notably, all compounds exhibited lower activity against the Panc-1 cancer cell line in a cell monolayer, but the effects on spheroid cell viability in 3D models were comparable across all tested cancer cell lines. Molecular docking studies of the most active hydrazones suggested that these compounds may act as multikinase inhibitors. In particular, 2-hydroxynaphthalenylmethylene derivative **12** showed high binding affinity values (−11.174 and −11.471 kcal/mol) to the active sites of two key protein kinases—a non-receptor TK (SCR) and STPK (BRAF)—simultaneously.

## 1. Introduction

Cancer is a complex and heterogeneous group of diseases characterized by the rapid, abnormal, and uncontrolled growth of cells [1]. Different types of cancer can vary substantially in their behavior and response to treatment [2]. In 2020, 18.1 million cancer cases were estimated worldwide, and it is estimated that the global cancer burden will increase by 47% by 2040 [3]. Despite advancements in research and treatment, cancer remains a significant global health challenge, requiring continued efforts toward innovative therapies. One of the most promising approaches in modern oncology is the targeted inhibition of key signaling proteins involved in tumor progression, particularly protein kinases, which regulate crucial cellular pathways in cancer development and metastasis [4].

Protein kinases catalyze the transfer of phosphate groups to target proteins, thereby modulating their activity [5]. Protein kinases are attractive targets for anticancer therapy, especially for treating melanoma, breast cancer, and pancreatic cancer, which are among the most aggressive types of cancer [6,7,8]. Targeted inhibition of these kinases holds promise in disrupting oncogenic signaling cascades, providing a tailored approach to cancer treatment [9].

In the context of melanoma, breast cancer, and pancreatic cancer, several types of kinase inhibitors have demonstrated efficacy in clinical settings [6,7,8]. Tyrosine kinase inhibitors targeting receptor and non-receptor tyrosine kinases [10] and serine/threonine protein kinase inhibitors [11] have shown remarkable success in halting tumor progression. In melanoma, aberrant activation of the BRAF/MEK signaling pathway plays a pivotal role in tumor progression, with mutations in the BRAF kinase being present in approximately 50% of melanomas, leading to the constitutive activation of the MAPK/ERK pathway [10,11]. This pathway promotes the uncontrolled proliferation and survival of melanoma cells, making BRAF and MEK inhibitors essential components of targeted melanoma therapy. In breast cancer, inhibitors targeting epidermal growth factor receptor (EGFR), human epidermal growth factor receptor 2 (HER2), and cyclin-dependent kinases (CDKs) have emerged as valuable therapeutic options [12,13]. Similarly, in pancreatic cancer, targeting receptor tyrosine kinases like HER2 and vascular endothelial growth factor receptor (VEGFR) has demonstrated clinical benefits [14]. Non-receptor tyrosine kinases like SRC have also gained attention for their roles in breast cancer and pancreatic cancer progression [15,16]. SRC is overexpressed in many cancers and is involved in the regulation of migration, invasion, and resistance to chemotherapy [14,15]. The nonreceptor tyrosine kinase ACK-1 has gained attention as a potential therapeutic target in triple-negative breast cancer [17,18] and pancreatic cancer [17,19], contributing to aggressive tumor behavior and a poor prognosis. While the development of ACK-1 inhibitors is still in its early stages, the promising preclinical data underscore the importance of continuing research in this area [20]. Given the crucial roles of these kinases in cancer progression, they have emerged as promising therapeutic targets for drug development.

However, all of the mentioned inhibitors face problems such as selectivity, toxicity, and resistance [21]. Understanding protein kinases’ involvement in melanoma, breast cancer, and pancreatic cancer could unveil new therapeutic possibilities, emphasizing the importance of continued research in identifying and developing innovative kinase inhibitors.

**Figure 1:**
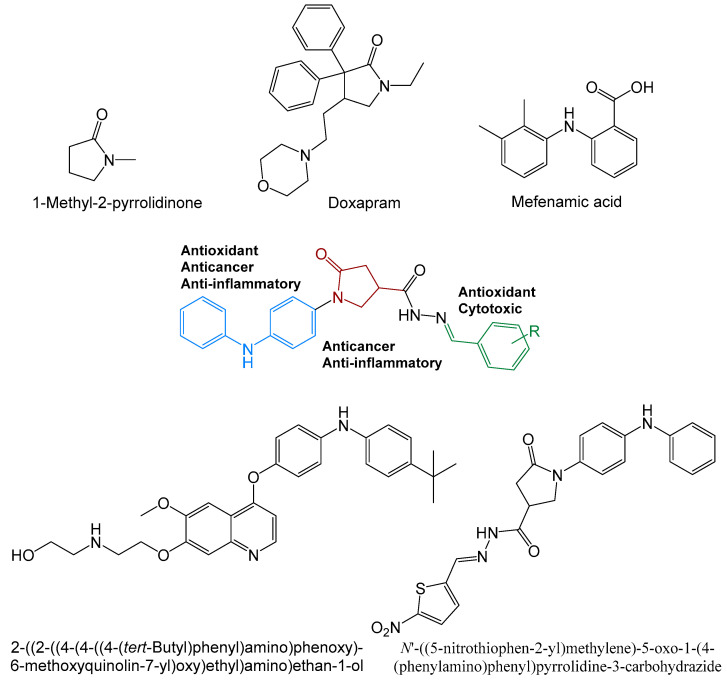
Commercial drugs and synthetic biologically active compounds [31,34] bearing 5-oxopyrrolidine, diphenylamine, and hydrazone moieties.

5-Oxopyrrolidines are one of the most important classes of biologically active *N*-heterocyclic compounds found in nature or synthesized in laboratories [22]. The 5-oxopyrrolidine scaffold is a constituent part of approved drugs [23,24]. 1-Methylpyrrolidinone is under investigation for the treatment of multiple myeloma [25] (Figure 1). The pyrrolidine moiety’s unconstrained conformation of the ring is beneficial in drug design, as it may be tuned and fixed with the selected substituents [26]. Furthermore, the 5-oxopyrrolidine moiety incorporated in hybrid chemical structures has exhibited anticancer activity [27,28,29].

Diphenylamine derivatives have been reported to possess different biological properties, such as analgesic, antimicrobial, anti-inflammatory [30], as well as anticancer properties [31,32]. A series of diphenylamines bearing methoxy substituents, among others, on one benzene ring were tested against five human cancer cell lines: MCF-7, MDA-MB-231, A549, HeLa, and HT29 cells. Their representative compound exhibited promising anticancer activity against the HT29 cell line [33].

Molecular docking studies play a crucial role in modern drug discovery and medicinal chemistry, providing a computational framework for understanding ligand–receptor interactions at the molecular level [35,36]. This approach accelerates the lead optimization process, saving time and resources before engaging in costly biological assays. By combining molecular docking data with experimental studies, researchers can improve the binding affinity and selectivity of new compounds [37,38].

Based on our continued interest in the search for the nitrogen-containing heterocyclic compounds possessing anticancer activity [34,39,40,41], we report the synthesis of a series of 5-oxopyrrolidine-hydrazone derivatives bearing a diphenylamine moiety, as well as in vitro and in silico evaluations of their anticancer potential.

## 2. Results and Discussion

### 2.1. Chemistry

Hydrazone derivatives represent an important class of bioactive compounds in medicinal and pharmaceutical chemistry, with their biological properties linked to the presence of the reactive azomethine pharmacophore [42]. These compounds, whose molecules comprise various heterocyclic scaffolds, exhibit different biological effects, including anticancer activities [43,44,45,46,47]. As a continuation of our interest in exploring anticancer activities of hydrazones bearing diphenylamine and 5-oxopyrrolidine moieties [34], this study focuses on hydroxy- and alkoxybenzene/naphthalene derivatives (Figure 1). It has been demonstrated that the anticancer activity of the molecules increases upon the introduction of hydroxyl and/or alkoxyl substituents into their structures [48,49].

**Scheme 1:**
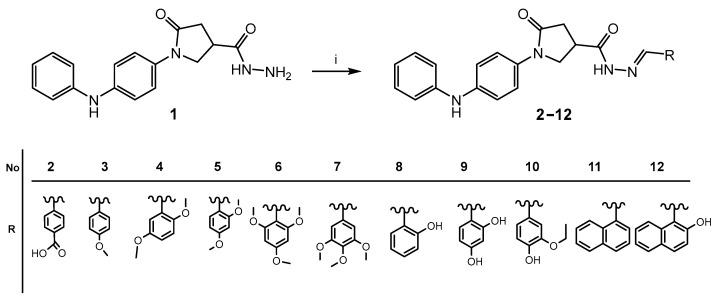
Synthesis of the target hydrazones **2**–**12**. Reagents and conditions: (i) corresponding aldehyde, MeOH, 60–70 °C 20 min–4 h., HCl to pH 6 (57–87%).

Hydrazones **2**–**12** were synthesized according to the synthesis pathway reported previously [39,40,41], as depicted in Scheme 1. Target hydrazones **2**–**12** were synthesized from 5-oxo-1-(4-(phenylamino)phenyl)pyrrolidine-3-carbohydrazide (**1**) [40] and the corresponding aldehydes (Scheme 1) in methanol at 60–70 °C in the presence of hydrochloric acid in a 57–87% yield.

The structures of the synthesized compounds were confirmed by ^1^H, ^13^C NMR, IR, and HRMS data (Supplementary Materials Figures S1–S33). The presence of the benzoic acid moiety in the structure of hydrazone **2** has was confirmed by the singlet at 13.06 ppm in the ^1^H NMR spectrum (Figure S1) and three carbon resonances in the region of 171–175 ppm attributed to the C=O groups in the ^13^C NMR spectrum (Figure S2). In the ^1^H NMR spectrum of *p*-methoxybenzene derivative **3** (Figure S4), the protons of the methyl group resonated as a singlet at 3.63 ppm, while the protons of two methoxy groups in compounds **4** and **5** gave two singlets at 3.78 and 3.79 ppm (**4**) and 3.65 and 3.69 ppm (**5**) in the respective ^1^H NMR spectra (Figures S7 and S10). In the ^1^H NMR spectra of compounds **6** and **7** (Figures S13 and S16), the protons of three methoxy groups resonated as three singlets at 3.76, 3.78, and 3.79 ppm; and 3.59, 3.62, and 3.80 ppm, respectively. The hydroxyl group proton in the ^1^H NMR spectrum for 2-hydroxybenzene derivative **8** gave two singlets at 11.07 ppm and 11.87 ppm, each with half intensity (0.5H) (Figure S19). The splitting of the resonances of the hydroxyl protons, as well as those of the NH group proton, is due to the existence of hydrazones as a mixture of *Z*/*E* isomers resulting from the hindered rotation around the amide bond in DMSO-*d_6_* solutions. In the ^1^H NMR spectra, the resonances attributed to the NH protons of the *Z* isomers are in a lower field compared to the signals of the same protons in the *E* isomers [50]. The same pattern of double sets of resonances attributed to the OH and NH group protons is observed in the ^1^H NMR spectra of 2,4-dihydroxybenzylidene derivative **9** (Figure S22) and 2-hydroxynaphthalenylmethylene derivative **12** (Figure S31).

### 2.2. Pharmacology

#### 2.2.1. The Compounds’ Effects on Cancer Cell Viability

**Figure 2:**
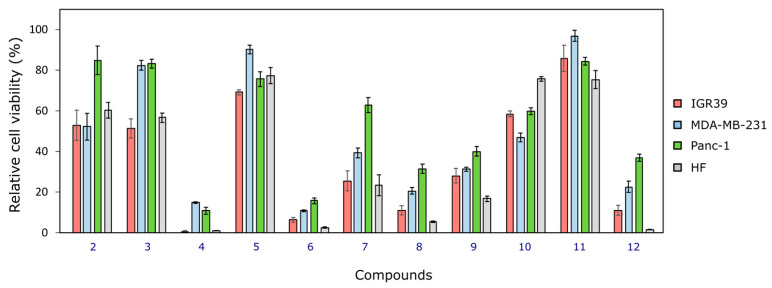
Effect of compounds **2**–**12** at a 100 µM concentration on the viability of human melanoma IGR39 cells, human triple-negative breast cancer MDA-MB-231 cells, human pancreatic carcinoma Panc 1 cells, and human fibroblasts, HFs, *n* = 3. The effect was established after 72 h of incubation by the MTT assay.

The cytotoxic activity of compounds **2**–**12** against the tested cancer cell lines differed (Figure 2). Five of the tested hydrazones (**2**, **3**, **5**, **10**, and **11**) showed very low activity against all cancer cell lines; they reduced cell viability only up to approx. 50% after 72 h of incubation. Compounds **7** and **9** were moderately active—the viability of all cell lines ranged from 20 to 70%. Compounds bearing the 2,4-dimethoxybenzylidene (**4**), 2,4,6-trimethoxybenzylidene (**6**), 2-hydroxybenzylidene (**8**), and 2-hydroxynaphthalenylmethylene (**12**) moieties were identified as the most active ones. In the presence of these compounds, the viability of cell lines was reduced to <20%. In addition, it was noted that most of the tested compounds showed relatively lower activity against the human pancreatic cancer cell line Panc-1 (Figure 2). This could be explained by the well-known high resistance of this cell line to various anticancer agents [51] and our previous observations [34].

The activity of the tested hydrazone derivatives **2**–**12** against melanoma IGR39 cells and the triple-negative breast cancer cell line MDA-MB-231 was similar and also varied among compounds. The most active compounds **4**, **6**, **8**, and **12** showed the strongest effects on melanoma cells (viability ranged from 0.2 to 11.3%) and were less cytotoxic toward MDA-MB-231 cells (viability ranged from 11.1 to 23.3%). However, it is worth noting that all of the most active compounds were not selective toward cancer cells when compared to human fibroblasts (normal cells).

**Figure 3:**
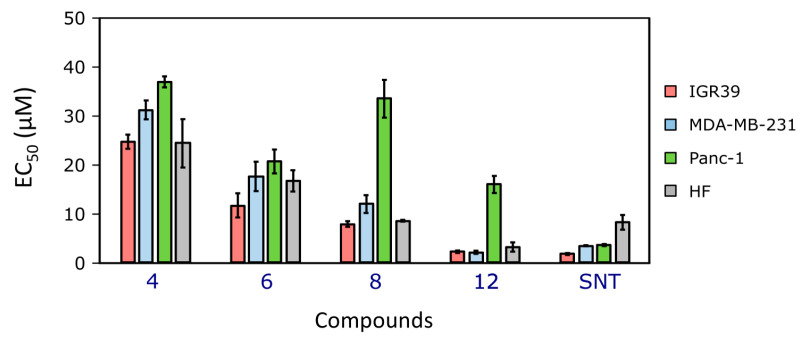
The EC_50_ values of selected compounds and sunitinib (SNT) in human melanoma IGR39, human triple-negative breast cancer MDA-MB-231, human pancreatic carcinoma Panc 1 cell lines, and human fibroblasts, HFs, *n* = 3. The effect was established after 72 h of incubation, by MTT assay.

Hydrazones **4**, **6**, **8**, and **12** were studied more thoroughly by determining their EC_50_ values (Figure 3). Generally, all four compounds showed the lowest activity against the human pancreatic carcinoma cell line Panc-1 (the EC_50_ ranged from 15.9 to 36.9 µM). Compared to the determined dose–response results in fibroblasts, none of the compounds exhibited selectivity toward pancreatic cancer cells. However, relative selectivity, or at least comparable activity, was established for compounds **4**, **6**, **8**, and **12** toward the human melanoma cell line IGR39, and for compounds **4**, **6,** and **12** toward the triple-negative breast cancer cell line MDA-MB-231. Compound **12** (bearing the 2-hydroxynaphthalenylmethylene moiety) was the most active against all tested cell lines (EC_50_ = 2.2 ± 0.3 µM against IGR39 cells, EC_50_ = 2.1 ± 0.3 µM against MDA-MB-231 cells, and EC_50_ = 15.9 ± 1.8 µM against the Panc-1 cell line).

Compounds **4, 6** and **8** were less active than the clinically used kinase inhibitor sunitinib (SNT). However, compound **12** was 1.7 times more cytotoxic than SNT toward the MDA-MB-231 cell line and exhibited similar activity against the melanoma IGR39 cell line, highlighting its potential for further development in the treatment of triple-negative breast cancer and melanoma. On the contrary, it was 2.5 times more cytotoxic than SNT and about 5 times less active against the pancreatic cancer cell line Panc-1. The established differences also indicate possible variations in the mechanism of action of compound **12** compared to SNT, necessitating further investigation to develop new candidates specific for different types of tumors. Additionally, all four compounds demonstrated higher cytotoxicity toward the melanoma cell line than the chemotherapeutic agent dacarbazine, which inhibits melanoma cell proliferation at concentrations of 25–100 μM [52]. On the other hand, some clinically used and even more active drugs, such as dabrafenib, are active in in vitro assays at nanomolar concentrations [53]. Moreover, despite the not particularly promising results regarding selectivity, it is noted that the cytotoxic effect on a cell monolayer is not necessarily translatable into in vivo results, as this simple model differs significantly from the real tumor microenvironment [54]. We have discussed the importance of the interpreting selectivity results in our previous publication [34], and considering its complexities and the potential misinterpretations involved, we decided to continue our experiments of the identified lead compounds by testing their cell migration-inhibiting effect and activity in a spheroid assay. In summary, the selection of the most promising candidates cannot be based solely on the EC_50_ values and selectivity, as there are many more important factors contributing to their suitability for clinical application, such as their pharmacokinetic properties, selective accumulation in tumors, etc. [55].

#### 2.2.2. The Compounds’ Effects on Cancer Cell Migration

**Figure 4:**
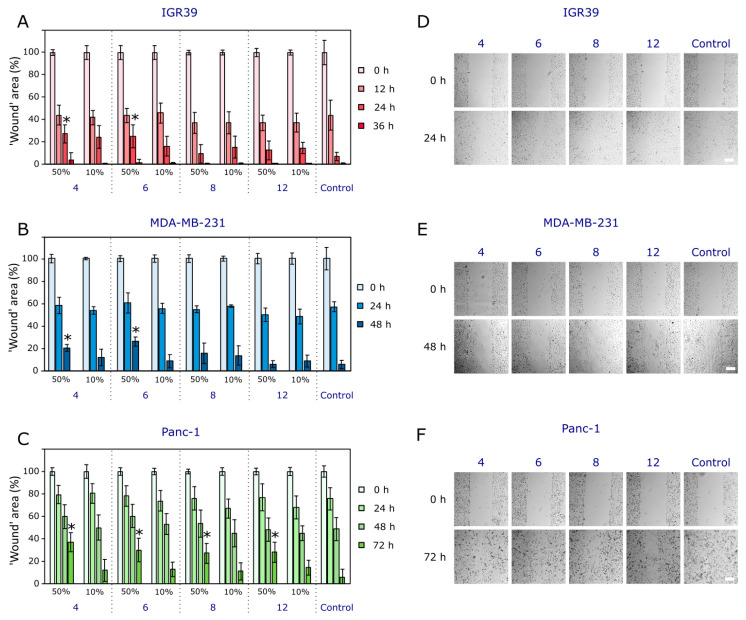
Effects of compounds **4**, **6**, **8**, and **12** on human malignant melanoma IGR39 (**A**), human triple-negative breast cancer MDA-MB-231 (**B**), and human pancreatic carcinoma (Panc-1) (**C**) cell migration, *n* = 3. Photos of the ‘wound’ areas in the IGR39 (**D**), MDA-MB-231 (**E**), and Panc-1 (**F**) monolayers at the beginning and the indicated time point of the experiment. The asterisk (*) indicates *p* < 0.05. The scale bar indicates 100 µm.

The effects of the most active hydrazones **4**, **6**, **8**, and **12** on cell migration were assessed using a ‘wound healing’ assay at concentrations of 10% and 50% of their EC_50_ values (Figure 4). These concentrations were chosen to compare the effects of these four compounds by avoiding possible effects on cell viability (at 10% of the EC_50_) or by at least maintaining the cytotoxic effect at a consistent level (50% of the EC_50_).

At the lowest concentration, there was no statistically significant effect of any compound on the migration of the tested cancer cell lines compared to the control (*p* > 0.05) (Figure 4A–C). Additionally, 2-hydroxybenzylidene derivative **8** and 2-hydroxynaphthalenylmethylene derivative **12** did not show an inhibitory effect on IGR39 and MDA-MB-231 cell migration at the higher concentration (Figure 4A,B). Meanwhile, all four compounds, at 50% of their EC_50_, statistically significantly reduced the migration of Panc-1 cells after 72 h of incubation (Figure 4C,F). A migration-inhibiting effect on IGR39 and MDA-MB-231 cells was established for 2,5-dimethoxybenzylidene derivative **4** and 2,4,6-trimethoxybenzylidene derivative **6**, but only after longer incubation periods (24 h and 48 h, respectively) (Figure 4A,B,D,E). The possible inhibition of kinases could explain such variable and delayed effects of the tested hydrazones on cell migration, and we noticed the same trend in our previous research [34]. Usually, such a phenomenon is caused by the changes in signaling pathways following kinase inhibition [56]. Also, it should be noted that the established effect at only the higher concentration and after a longer incubation could be partly the consequence of the concurrently reduced cell proliferation. The ‘wound healing’ assay is often criticized due to its problems related to reproducibility and the phenomenon of cell injury during scratching [57]. However, it is still widely used due to its simplicity as a preliminary evaluation of the compound’s possible ability to reduce cell migration. Thus, at this stage, we aimed to identify the compounds’ properties in affecting migration and later continue more detailed tests only with the most promising candidates.

#### 2.2.3. The Compounds’ Effects on Cancer Cell 3D Models (Cancer Spheroids)

Further experiments were performed in cell 3D cultures to determine the compounds’ effects on spheroid growth and viability. Based on our previous experience, we formed spheroids from cancer cells combined with fibroblasts in a 1:1 ratio. Fibroblasts are known to contribute to a better representation of the real tumor microenvironment [58], and play a crucial role in the formation of the 3D cultures by acting as supporting (stromal) cells.

**Figure 5:**
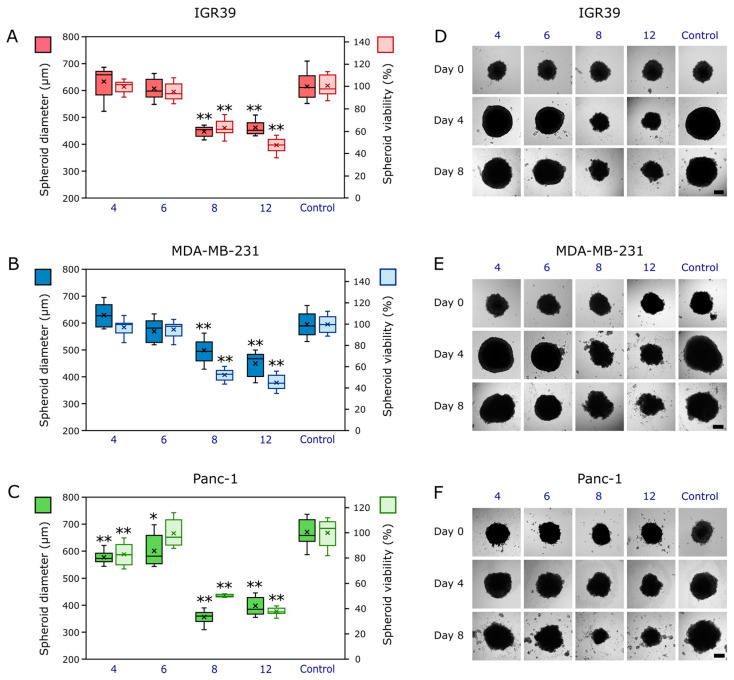
Impacts of the most potent compounds (**4**, **6**, **8**, and **12**) on cancer spheroids. (**A**) Spheroid size (brighter red) and cell viability within spheroids (lighter red) of human melanoma IGR39 cells on the last day of the experiment. (**B**) Spheroid size (brighter blue) and cell viability (lighter blue) in human triple-negative breast cancer MDA-MB-231 spheroids on the last day of the experiment. (**C**) Spheroid size (brighter green) and cell viability (lighter green) in human pancreatic carcinoma Panc-1 spheroids. (**D**) Representative images of IGR39 spheroids taken at the start (Day 0), midpoint (Day 4), and the end (Day 8) of treatment with 20 µM of the compounds. (**E**) Representative images of MDA-MB-231 spheroids taken at the start (Day 0), midpoint (Day 4), and the end (Day 8) of treatment with 20 µM of the compounds. (**F**) Panc-1 spheroid photographs taken on Days 0, 4, and 8, following exposure to 20 µM of the compounds. Statistical significance: (*) denotes *p* < 0.05 versus untreated controls, (**) indicates *p* < 0.01 versus controls. Crosses (×) represent mean values, inner dashes indicate medians, and whiskers illustrate maximum and minimum values. Scale bars = 200 µm.

At the initial stage of the experiment, both types of spheroids measured 300–400 µm in diameter (Figure 5D,E,F). It was noted that spheroids grew faster up to Day 4, and later, their size increased only slightly (in the case of Panc-1 spheroids) or even decreased (IGR39 and MDA-MB-231 spheroids). Considering the starting size of spheroids, it could be hypothesized that they had a hypoxic center inside [59], which contributed to their resemblance to the real tumor situation. Overall, spheroid growth was affected differently by incubation with the compounds, but a similar trend was observed across all spheroid types. Hydrazones **8** and **12** were the most active against 3D cultures of all cancer cell lines and reduced the viability of spheroid cells (Figure 5). Compound **4** was also active against Panc-1 spheroids and showed effects both on spheroid growth and viability, while compound **8** slightly inhibited the growth of Panc-1 spheroids but did not reduce their viability. This phenomenon has been previously observed by us and other researchers, as it is well known that the spheroid size and cell viability do not always correlate [60].

In summary, the most active compounds **4**, **6**, **8**, and **12** exhibited distinct anticancer properties. While compounds **8** and **12** demonstrated higher cytotoxicity in both 2D and 3D assays, compounds **4** and **6** exhibited greater effects on inhibiting cell migration. Moreover, generally, all compounds were less active against the Panc-1 cancer cell line in the cell monolayer. Still, the effect on spheroid cell viability in 3D cultures was quite similar between tested cancer cell lines. In addition, all compounds inhibited Panc-1 cell migration after a longer incubation (72 h). However, we acknowledge the limitations of the migration assay, as after 72 h, the cytotoxicity of the compounds could be the factor that contributed to slower ‘wound area’ closure.

### 2.3. Molecular Docking Study

Based on the experimental data on antitumor activity against human melanoma IGR39, human triple-negative breast cancer (MDA-MB-231), and pancreatic carcinoma (Panc-1) cell lines, promising hydrazones **4**, **6**, **8**, and **12** were identified. To investigate the potential mechanism of the anticancer activity of the synthesized compounds, molecular docking studies were conducted. The kinases chosen for docking were selected based on their known involvement in our target cancers [6,7,8,9,10,11,12,13,14,15,16] and the structural features of the investigated compounds. BRAF and MEK1/2 were included due to the central role of the MAPK pathway in melanoma (activating BRAF^V600E mutations occur in ~50% of melanomas). Targeting BRAF/MEK has proven therapeutic relevance in melanoma, as the combined inhibition of these kinases yields a marked clinical benefit [61]. In triple-negative breast cancer (TNBC), the focus was on EGFR and Src because EGFR is frequently overexpressed in TNBC tumors [62] and Src-mediated signaling drives TNBC cell survival and invasiveness [63]. ACK1 was examined as it is aberrantly activated in breast and pancreatic cancers, activating pro-survival pathways [64]. We also evaluated VEGFR2, given that TNBC and pancreatic cancers are highly dependent on angiogenesis [65]. CDK5 was included based on emerging evidence that it facilitates pancreatic cancer invasiveness. CDK5 and its activators are amplified in a majority of pancreatic tumors [66]. Importantly, the 5-oxopyrrolidine-3-carbohydrazide structure of the investigated compounds shares key pharmacophores with known kinase inhibitors. The aryl moiety in our molecules is a classical motif present in many ATP-competitive inhibitors [67] such as dasatinib, which targets SRC and ACK-1, and vemurafenib, a BRAF inhibitor containing a substituted arylamide core capable of hinge region interactions. In addition, the 5-oxopyrrolidine ring in the investigated compounds serves as a structural analogue to the pyrrolopyridine core of kinase inhibitors like vemurafenib, and shares pharmacophoric similarities with heterocyclic systems in trametinib, allowing for potential hydrogen bonding via its lactam NH and C=O groups at the ATP-binding site or adjacent allosteric regions. Likewise, the acylhydrazide linker in our compounds can mimic the hydrogen-bonding roles of urea or amide linkers (enhancing binding to the kinase hinge region) [68]. These parallels suggest that our novel compounds can engage the ATP-binding sites of the selected kinases, justifying our docking-driven target selection.

Therefore, we included receptor tyrosine kinases EGFR (PDB: 1M17, 4HJO, 1XKK), VEGFR (PDB: 4AGD, 4ASD, 3EWH), HER2 (PDB: 3RCD, 3PPO), non-receptor tyrosine kinases SCR (PDB: 1A07, 3F3V), ACK-1 (PDB: 5ZXB), and serine/threonine protein kinases (STPKs) BRAF (PDB: 4RZV, 7MOU), MEK (PDB: 4U7Z, 7MOY), and CDK5 (PDB: 1UNL) that are frequently overactivated or mutated in these cancer types.

The docking study results demonstrated the high affinity of the investigated compounds **4**, **6**, **8**, and **12** for the non-receptor tyrosine kinases ACK-1 and SCR, as well as for the serine threonine protein kinases BRAF and MEK (Table 1). The following FDA-approved multitarget drugs used in the treatment of melanoma, breast cancer, and pancreatic cancer, respectively, were used as reference ligands: vemurafenib (BRAF inhibitor) and trametinib (MEK inhibitor) for melanoma, and dasatinib (ACK-1 and SCR inhibitor) for breast cancer and pancreatic cancer.

According to the results presented in Table 1, compounds **6** (ACK-1), **8** (MEK), and **12** (SCR and BRAF) showed the best binding in the docking score and Glide Emodel parameters compared to standard ligands. In all cases, these compounds outperformed the reference drugs in terms of the docking score parameter. Additionally, based on the Glide Emodel parameter, their values were comparable to or exceeded those of the reference ligands.

**Figure 6:**
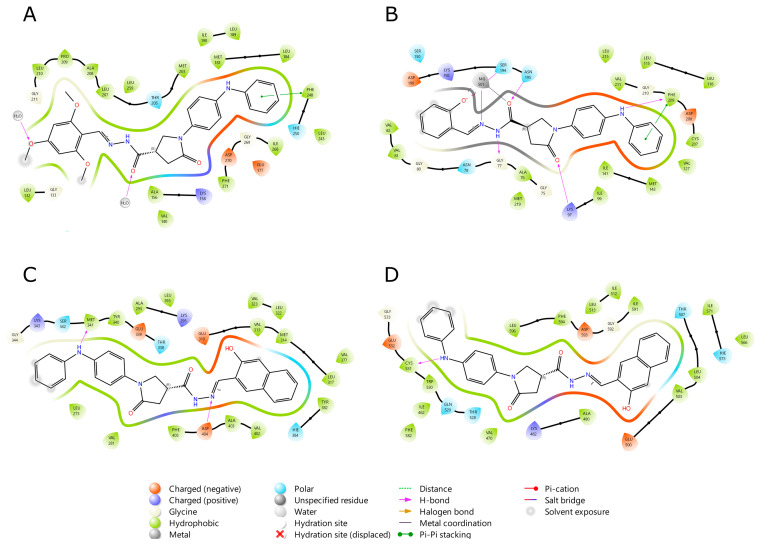
Two-dimensional views of the interactions of the compounds with amino acid residues in the active sites of the corresponding proteins: (**A**)—compound **6** with ACK-1 (5ZXB), (**B**)—compound **8** with MEK (4U7Z), (**C**)—compound **12** with SCR (3F3V), and (**D**)—compound **12** with BRAF (1UWH).

**Figure 7:**
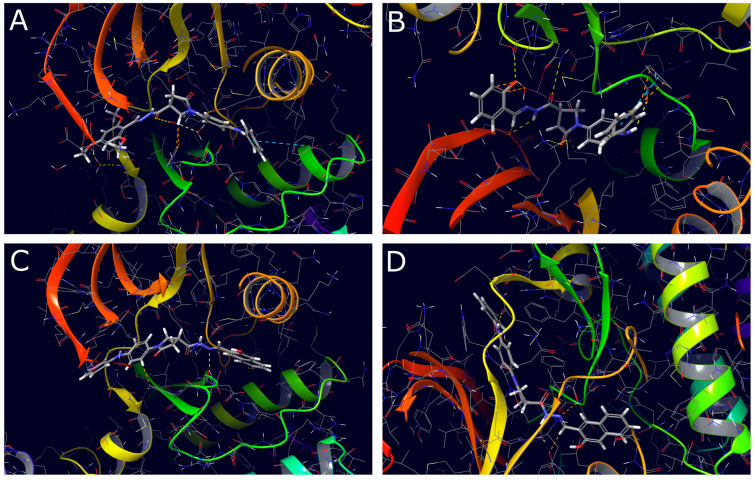
Three-dimensional views of the interactions of the compounds with amino acid residues in the active sites of the corresponding proteins: (**A**)—compound **6** with ACK-1 (5ZXB), (**B**)—compound **8** with MEK (4U7Z), (**C**)—compound **12** with SCR (3F3V), and (**D**)—compound **12** with BRAF (1UWH).

For compounds **6** and **8**, which exhibited high affinity for non-receptor tyrosine kinases, a π-π stacking interaction between the phenylalanine residue and the phenyl ring of the molecules was observed. Molecule **8** was stabilized in the hydrophobic pocket by means of five hydrogen bonds with hydrophobic, positively charged, polar amino acid residues (Figure 6 and Figure 7). Compound **12** displayed high affinity values (−11.174 and −11.471 kcal/mol) for the active sites of two types of protein kinases simultaneously—a non-receptor TK (SCR) and STPK (BRAF). This may indicate a dual mechanism of kinase inhibition for this molecule.

An analysis of the docking score values obtained for the compounds **4**, **6**, **8**, and **12** (Table 1) suggests that these compounds may act as multikinase inhibitors.

The correlation between the experimental results and the molecular docking findings revealed a strong alignment. The biological findings for compounds **4**, **6**, **8**, and **12** correspond with the molecular docking results, which identified BRAF, MEK, SRC, and ACK-1 as the most likely molecular targets of the active compounds. Compounds **8** and **12** exhibited the strongest cytotoxicity and spheroid inhibition, consistent with their high docking scores for BRAF and SRC. The strong binding of compound **12** to both kinases suggests that its effectiveness could be due to the simultaneous inhibition of MAPK/ERK signaling (BRAF) and invasion/metastasis (SRC), explaining its superior performance in both 2D and 3D models. Compounds **4** and **6** significantly inhibited cancer cell migration, correlating with their interactions with ACK-1 and MEK, both of which are involved in cancer cell invasion and proliferation. Compound **8** exhibited potent cytotoxic effects and MEK inhibition, aligning with its high docking score for MEK (−10.885 kcal/mol).

We analyzed the specific amino acid residues within the kinase active sites that participate in key interactions with the synthesized hydrazone derivatives **6**, **8**, and **12** (Figure 6 and Figure 7). The following critical residues were identified as forming hydrogen bonds, π-π stacking interactions, or hydrophobic interactions. The ligand interaction for compound **6** reveals that amino acid residue Phe248 forms a π-π stacking interaction with the aromatic ring of the ligand (Figure 6A and Figure 7A). Hydrazone **6** is located in a hydrophobic pocket, which is formed by the amino acid residues leucine (Leu184, Leu132, Leu210, and Leu207), phenylalanine (Phe248 and Phe271), isoleucine (Ile268), alanine (ALA208 and Ala156), methionine (Met 181 and Met203), proline (Pro209), glycine (Gly269 and Gly211) and negatively charged aspartic acid (Asp270). Compound **8**, in addition to the π-π stacking interaction of Phe209 residue with the aromatic ring of the ligand, forms an H-bond with the nitrogen atom of the secondary amino group (Figure 6B and Figure 7B). Hydrogen bonds are also formed between amino acid residues Gly77, positively charged Lys97, and polar Asn195 with ligand **8**. Key interactions of compound **12** (Figure 6C and Figure 7C) with BRAF (PDB: 1UWH) include negatively charged Glu500, Asp593, hydrophobic Phe594, positively charge Lys482, and Cys531 (H-bond). Key interactions of compound **12** (Figure 6D and Figure 7D) with SRC (PDB: 3F3V) include hydrogen bonding with an ylidene nitrogen atom in the hydrazone fragment (negatively charged Asp404) and the secondary amino group (hydrophobic Met341). Therefore, the interactions mentioned above demonstrate their significant roles in the binding affinity of derivatives **6**, **8**, and **12** to the active sites of the corresponding proteins.

These findings show our molecular docking results as a useful predictor of biological activity and highlight the therapeutic potential of the synthesized hydrazones as multi-kinase inhibitors. Future studies will focus on kinase inhibition assays to confirm these computational predictions and further elucidate the mechanisms of action.

## 3. Materials and Methods

### 3.1. Chemistry

Reagents were purchased from Sigma-Aldrich (St. Louis, MO, USA) and TCI Europe N.V. (Zwijndrecht, Belgium). The reaction course and purity of the synthesized compounds were monitored by TLC using aluminum plates precoated with silica gel 60 F254 (MerckKGaA, Darmstadt, Germany). The ^1^H and ^13^C NMR spectra were recorded in DMSO-*d_6_* on a Bruker Avance III (400 MHz, 101 MHz) spectrometer (Bruker BioSpin AG, Fällanden, Switzerland) operating in the Fourier transform mode. Chemical shifts (*δ*) are reported in parts per million (ppm) calibrated from TMS (0 ppm) as an internal standard for ^1^H NMR and DMSO-*d_6_* (39.43 ppm) for ^13^C NMR. FT-IR spectra (ν, cm^−1^) were recorded on a Perkin–Elmer Spectrum BX FT–IR spectrometer (Perkin–Elmer Inc., Waltham, MA, USA) using KBr pellets. Mass spectra were obtained on a Bruker maXis UHR-TOF mass spectrometer (Bruker Daltonics, Bremen, Germany) with positive ESI ionization. The melting points were determined on a MEL-TEMP (Electrothermal, A Bibby Scientific Company, Burlington, NJ, USA) melting point apparatus and are uncorrected.

5-Oxo-1-(4-(phenylamino)phenyl)pyrrolidine-3-carbohydrazide (**1**) was synthesized as described in [40]. M.p., ^1^H and ^13^C NMR spectra were found to be identical with the ones described in [40].

#### General Procedure for the Synthesis of Compounds **2**–**12**

To hydrazide **1** (1.5 mmol) dissolved in methanol (25 mL), a corresponding aldehyde (2.5 mmol) was added, followed by addition of concentrated HCl (2–3 drops). The reaction mixture was stirred at 60–70 °C for 20 min–4 h. The precipitate formed was filtered off, dried and recrystallized from the DMF/H_2_O mixture.

4-((2-(5-Oxo-1-(4-(phenylamino)phenyl)pyrrolidine-3-carbonyl)hydrazono)methyl)benzoic acid (**2**) (E/Z isomeric mixture in DMSO-*d_6_* solution).

Prepared from 4-carboxybenzaldehyde. Yield 0.74 g (73%), grey crystals, m.p. 262–263 °C; TLC: *R*_f_ = 0.11 (acetone/hexane = 1:1); IR (KBr) ν_max_ (cm^−1^): 1563, 1602, 1671 (C=O), 3037, 3222 (NH), 3392 (OH); ^1^H NMR (400 MHz, DMSO-*d_6_*): *δ* = 2.69–2.83 (m, 2H, H_14_), 4.04–4.11 (m, 3H, H_15,16_), 6.76 (t, 1H, *J* = 7.6 Hz, H_4_), 7.01–7.08 (m, 4H, H_2,6,8,12_), 7.19 (t, 2H, *J* = 8.0 Hz, H_3,5_), 7.46 (d, 2H, *J* = 8.4 Hz, H_9,11_), 7.78–7.98 (m, 4H, H_20,21,23,24_), 8.07 (s, 1H, NH), 8.23 (s, 0.6H, H_18_), 8.72 (s, 0.4H, H_18_), 11.62 (s, 0.6H, NH), 11.84 (s, 0.4H, NH), 13.06 (s, 1H, OH); ^13^C NMR (101 MHz, DMSO-*d_6_*): *δ* = 33.2 (C_15_), 35.3 (C_14_), 50.8 (C_16_), 116.7, 117.5, 120.0, 121.7, 127.3 127.6, 128.9, 129.6, 130.2, 131.9, 133.4, 137.8, 138.5, 140.4, 143.2, 143.9, 146.5, 161.4, 167.4 (C_Ar,Ar′,Ar″_+C_18_), 171.8, 172.0, 174.4 (C_13,17,25_). HRMS (ESI+): *m*/*z* calcd for C_25_H_23_N_4_O_4_ 443.172 [M+H]^+^, found 443.1718.

*N’*-(4-methoxybenzylidene)-5-oxo-1-(4-(phenylamino)phenyl)pyrrolidine-3-carbohydrazide (**3**) (E/Z isomeric mixture in DMSO-*d_6_* solution).

Prepared from 4-methoxybenzaldehyde. Yield 0.67 g (68%), pink crystals, m.p. 240–241 °C; TLC: *R*_f_ = 0.1 (acetone/hexane = 1:1); IR (KBr) ν_max_ (cm^−1^): 1603, 1678 (C=O), 2927, 3318 (NH); ^1^H NMR (400 MHz, DMSO-*d_6_*): *δ* = 2.65–2.84 (m, 2H, H_14_), 3.63 (s, 3H, H_25_), 3.89–3.93 (m, 1H, H_15_), 3.99–4.12 (m, 2H, H_16_), 6.79 (t, 1H, J = 7.2 Hz, H_4_), 7.00–7.07 (m, 4H, H _2,6,8,12_), 7.18–7.21 (m, 2H, H_3,5_), 7.46 (d, 2H, J = 7.2 Hz, H_9,11_), 7.59–7.65 (m, 4H, H_Ar″_), 7.99 (s, 0.6H, H_18_), 8.12 (s, 1H, NH), 8.15 (s, 0.4H, H_18_), 11.54 (s, 0.6H, NH), 11.76 (s, 0.4H, NH); ^13^C NMR (101 MHz, DMSO-*d_6_*): *δ* = 32.9 (C_15_), 35.8 (C_14_), 50.0 (C_16_), 55.4 (C_25_), 113.6, 114.4, 116.4, 116.8, 121.1, 121.2, 125.8, 126.6, 128.4, 129.6, 130.0, 136.8, 141.6, 143.5, 145.8, 160.5, 161.7, 162.3, 169.6 (C_Ar,Ar′,Ar″_+C_18_), 173.2, 175.7 (C_13,17_). HRMS (ESI+): *m*/*z* calcd for C_25_H_25_N_4_O_3_ 429.1927 [M+H]^+^, found 429.1923.

*N′*-(2,5-dimethoxybenzylidene)-5-oxo-1-(4-(phenylamino)phenyl)pyrrolidine-3-carbohydrazide (**4**) (E/Z isomeric mixture in DMSO-*d_6_* solution)

Prepared from 2,5-dimethoxybenzaldehyde. Yield 0.92 g (87%), blue crystals, m.p. 190–191 °C; TLC: *R*_f_ = 0.23 (acetone/hexane = 1:1); IR (KBr) ν_max_ (cm^−1^): 1665, 1696 (C=O), 2938, 3389 (NH); ^1^H NMR (400 MHz, DMSO-*d_6_*): *δ* = 2.65–2.86 (m, 2H, H_14_), 3.78, 3.79 (2s, 6H, H_25,26_), 3.92–3.95 (m, 1H, H_15_), 4.00–4.11 (m, 2H, H_16_), 6.78 (t, 1H, *J* = 7.6 Hz, H_4_), 6.97 (s, 1H, H_24_), 7.00–7.21 (m, 8H, H_2,3,5,6,8,12,21,22_), 7.47 (d, 2H, *J* = 8.8 Hz, H_9,11_), 7.94 (s, 0.5H, H_18_), 8.10 (s, 1.5H, H_18_+NH), 11.48 (s, 0.5H, NH), 11.70 (s, 0.5H, NH); ^13^C NMR (101 MHz, DMSO-*d_6_*): *δ* = 33.3 (C_15_), 35.2, 35.8 (C_14_), 51.0, 51.3 (C_16_), 56.3, 60.5 (C_25,26_), 104.5, 104.8, 116.7, 117.5, 120.0, 121.7, 129.6, 130.0, 131.9, 132.0, 139.4, 139.6, 140.4, 143.9, 144.1, 147.6, 153.5, 169.5 (C_Ar,Ar′,Ar″_+C_18_), 171.8, 174.2 (C_13,17_). HRMS (ESI+): *m*/*z* calcd for C_26_H_27_N_4_O_4_ 459.2033 [M+H]^+^, found 459.1853.

*N′*-(2,4-dimethoxybenzylidene)-5-oxo-1-(4-(phenylamino)phenyl)pyrrolidine-3-carbohydrazide (**5**) (E/Z isomeric mixture in DMSO-*d_6_* solution).

Prepared from 2,4-dimethoxybenzaldehyde. Yield 0.77 g (73%), yellow crystals, m.p. 206–207 °C; TLC: *R*_f_ = 0.34 (acetone/hexane = 1:1); IR (KBr) ν_max_ (cm^−1^): 1605, 1680 (C=O), 2934, 3384 (NH); ^1^H NMR (400 MHz, DMSO-*d_6_*): *δ* = 2.70–2.75 (m, 2H, H_14_), 3.65, 3.69 (2s, 6H, H_25,26_), 3.95–4.06 (m, 3H, H_15,16_), 5.54 (s, 1H, NH), 6.45–6.53 (m, 1H, H_4_), 6.56–6.61 (m, 2H, H_24,25_), 6.85–6.87 (m, 2H, H_2,6_), 6.92–6.96 (m, 2H, H_8,12_), 7.00–7.05 (m, 2H, H_3,5_), 7.38–7.45 (m, 2H, H_9,11_), 7.72–7.77 (m, 1H, H_21_), 8.26 (s, 0.6H, H_18_), 8.44 (s, 0.4H, H_18_), 11.32 (s, 0.6H, NH), 11.55 (s, 0.4H, NH); ^13^C NMR (101 MHz, DMSO-*d_6_*): *δ* = 33.3 (C_15_), 35.8 (C_14_), 50.9 (C_16_), 55.4, 55.8 (C_25,26_), 98.8, 104.64, 106.8, 115.3, 116.7, 117.0, 121.7, 127.3, 130.0, 131.6, 136.0, 140.1, 140.7, 141.7, 143.3, 157.7, 159.3, 159.4, 159.6, 162.7, 162.9, 169.0 (C_Ar,Ar′,Ar″_+C_18_), 171.8, 173.8 (C_13,17_). HRMS (ESI+): *m*/*z* calcd for C_26_H_27_N_4_O_4_ 459.2033 [M+H]^+^, found 459.1849.

5-Oxo-1-(4-(phenylamino)phenyl)-*N′*-(2,4,6-trimethoxybenzylidene)pyrrolidine-3-carbohydrazide (**6**) (E/Z isomeric mixture in DMSO-*d_6_* solution).

Prepared from 2,4,6-trimethoxybenzaldehyde. Yield 0.95 g (85%), blue crystals, m.p. 215–216 °C; TLC: *R*_f_ = 0.47 (acetone/hexane = 1:1); IR (KBr) ν_max_ (cm^−1^): 1651, 1687 (C=O), 3105, 3403 (NH); ^1^H NMR (400 MHz, DMSO-*d_6_*): *δ* = 2.68–2.77 (m, 2H, H_14_), 3.76, 3.78, 3.79 (3s, 9H, H_25,26,27_), 3.86–4.11 (m, 3H, H_15,16_), 6.24 (s, 2H, H_21,23_), 6.78 (t, 1H, *J* = 7.2 Hz, H_4_), 7.02 (d, 2H, *J* = 8.0 Hz, H_2,6_), 7.06 (d, 2H, *J* = 8.4 Hz, H_8,12_), 7.20 (t, 2H, *J* = 7.6 Hz, H_3,5_), 7.43–7.47 (m, 2H, H_9,11_), 8.11 (s, 1H, NH), 8.18 (s, 0.7H, H_18_), 8.33 (s, 0.3H, H_18_), 11.14 (s, 0.7H, NH), 11.31 (s, 0.3H, NH); ^13^C NMR (101 MHz, DMSO-*d_6_*): *δ* = 34.2 (C_15_), 35.2 (C_14_), 50.9 (C_16_), 55.8 56.3, 56.3 (C_25,26,27_), 91.4, 104.1, 116.7, 117.5, 120.0, 121.6, 121.8, 129.6, 131.9, 132.0, 139.5, 140.4, 143.9, 160.2, 160.3, 162.5, 162.8, 168.6 (C_Ar,Ar′,Ar″_+C_18_), 171.9, 173.5 (C_13,17_). HRMS (ESI+): *m*/*z* calcd for C_27_H_29_N_4_O_5_ 489.2139 [M+H]^+^, found 489.2140.

5-Oxo-1-(4-(phenylamino)phenyl)-*N*′-(3,4,5-trimethoxybenzylidene)pyrrolidine-3-carbohydrazide (**7**) (E/Z isomeric mixture in DMSO-*d_6_* solution).

Prepared from 3,4,5-trimethoxybenzaldehyde. Yield 0.76 g (68%), dark green crystals, m.p. 220–221 °C; TLC: *R*_f_ = 0.16 (acetone/hexane = 1:1); IR (KBr) ν_max_ (cm^−1^): 1604, 1684 (C=O), 2937, 3325 (NH); ^1^H NMR (400 MHz, DMSO-*d_6_*): *δ* = 2.64–2.77 (m, 2H, H_14_), 3.59, 3.62, 3.80 (3s, 9H, H_25,26,27_), 3.97–4.07 (m, 3H, H_15,16_), 6.74 (t, 1H, *J* = 7.4 Hz, H_4_), 6.89 (d, 2H, *J* = 7.2 Hz, H_8,12_), 6.96 (d, 2H, *J* = 7.4 Hz, H_2,6_), 7.02 (d, 2H, *J* = 7.4 Hz, H_3,5_), 7.08 (s, 2H, H_20,24_) 7.40–7.46 (m, 2H, H_9,11_), 8.31 (s, 0.6H, H_18_), 8.49 (s, 0.4H, H_18_), 8.89 (s, 1H, NH), 11.48 (s, 0.6H, NH), 11.71 (s, 0.4H, NH); ^13^C NMR (101 MHz, DMSO-*d_6_*): *δ* = 33.3 (C_15_), 35.3 (C_14_), 51.2 (C_16_), 55.5, 55.8, 56.7 (C_25,26,27_), 110.2, 114.0, 116.7, 117.1, 117.3, 117.5, 119.9, 121.7, 122.3, 129.6, 130.0, 135.3, 141.8, 152.5, 152.7, 153.5, 153.8, 157.0, 169.32 (C_Ar,Ar′,Ar″_+C_18_), 171.6, 173.8 (C_13,17_). HRMS (ESI+): *m*/*z* calcd for C_27_H_29_N_4_O_5_ 489.2139 [M+H]^+^, found 489.1853.

*N′*-(2-hydroxybenzylidene)-5-oxo-1-(4-(phenylamino)phenyl)pyrrolidine-3-carbohydrazide (**8**) (E/Z isomeric mixture in DMSO-*d_6_* solution).

Prepared from 2-hydroxybenzaldehyde. Yield 0.77 g (81%), brown crystals, m.p. 210–211 °C; TLC: *R*_f_ = 0.29 (acetone/hexane = 1:1); IR (KBr) ν_max_ (cm^−1^): 1666, 1689 (C=O), 3032, 3358 (NH), 3405 (OH); ^1^H NMR (400 MHz, DMSO-*d_6_*): *δ* = 2.70–2.83 (m, 2H, H_14_), 3.94–3.98 (m, 1H, H_15_), 4.01–4.12 (m, 2H, H_16_), 6.79 (t, 1H, *J* = 6.8 Hz, H_4_), 6.84–6.95 (m, 2H, H_8,12_), 7.01–7.05 (m, 2H, H_2,6_), 7.07–7.10 (m, 2H, H_3,5_), 7.21 (t, 2H, *J* = 7.8 Hz, H_22,23_), 7.27–7.31 (m, 2H, H_9,11_), 7.49–7.55 (m, 2H, H_21,24_), 8.15 (s, 1H, NH), 8.36 (s, 0.4H, H_18_), 8.44 (s, 0.6H, H_18_), 10.07 (s, 0.4H, NH), 10.07 (s, 0.4H, NH), 10.26 (s, 0.2H, NH), 11.07 (s, 0.5H, OH), 11.51 (s, 0.4H, NH), 11.87 (s, 0.5H, OH); ^13^C NMR (101 MHz, DMSO-*d_6_*): *δ* = 32.9 (C_15_), 34.7 (C_14_), 50.4 (C_16_), 116.2, 117.2, 119.4, 121.0, 121.1 125.2, 125.7, 127.3, 127.5, 129.2, 129.9, 130.6, 131.9, 139.8, 140.7, 143.3, 143.7, 146.6, 168.7 (C_Ar,Ar′,Ar″_+C_18_), 171.4, 173.5 (C_13,17_). HRMS (ESI+): *m*/*z* calcd for C_24_H_23_N_4_O_3_ 415.1771 [M+H]^+^, found 415.1765.

*N′*-(2,4-dihydroxybenzylidene)-5-oxo-1-(4-(phenylamino)phenyl)pyrrolidine-3-carbohydrazide (**9**) (E/Z isomeric mixture in DMSO-*d_6_* solution).

Prepared from 2,4-dihydroxybenzaldehyde. Yield 0.66 g (67%), light blue crystals, m.p. 231–232 °C; TLC: *R*_f_ = 0.14 (acetone/hexane = 1:1); IR (KBr) ν_max_ (cm^−1^): 1630, 1669 (C=O), 3031, 3211 (NH), 3464, 3842 (OH); ^1^H NMR (400 MHz, DMSO-*d_6_*): *δ* = 2.63–2.81 (m, 2H, H_14_), 3.89–3.93 (m, 1H, H_15_), 3.98–4.06 (m, 2H, H_16_), 6.32 (d, 2H, *J* = 8.0 Hz, H_23,24_), 6.36–6.41 (m, 1H, H_21_), 6.79 (t, 1H, *J* = 7.4 Hz, H_4_), 7.02 (d, 2H, *J* = 8.0 Hz, H_8,12_), 7.06–7.08 (m, 2H, H_2,6_), 7.20 (t, 2H, *J* = 7.4 Hz, H_3,5_), 7.28–7.42 (m, 2H, H_9,11_), 8.20 (s, 0.4H, H_18_), 8.26 (s, 0.6H, H_18_), 8.73 (s, 1H, NH), 10.18 (s, 0.6H, OH); 10.42 (s, 0.4H, OH), 11.28 (s, 0.6H, NH), 11.73 (s, 0.4H, NH); ^13^C NMR (101 MHz, DMSO-*d_6_*): *δ* = 33.3 (C_15_), 35.4 (C_14_), 51.3 (C_16_), 102.9, 108.3, 108.8, 110.7, 110.8, 116.8, 117.6, 120.1, 121.8, 129.7, 131.8, 131.9, 133.6, 140.5, 143.9, 149.0, 159.8, 161.1, 162.5, 168.9 (C_Ar,Ar′,Ar″_+C_18_), 171.9, 173.9 (C_13,17_). HRMS (ESI+): *m*/*z* calcd for C_24_H_23_N_4_O_4_ 431.1720 [M+H]^+^, found 431.1717.

*N′*-(3-ethoxy-4-hydroxybenzylidene)-5-oxo-1-(4-(phenylamino)phenyl)pyrrolidine-3-carbohydrazide (**10**) (E/Z isomeric mixture in DMSO-*d_6_* solution).

Prepared from 3-ethoxy-4-hydroxybenzaldehyde. Yield 0.72 g (68%), light yellow crystals, m.p. 220–222 °C; TLC: *R*_f_ = 0.17 (acetone/hexane = 1:1); IR (KBr) ν_max_ (cm^−1^): 1653, 1678 (C=O), 2971, 3224 (NH), 3348 (OH); ^1^H NMR (400 MHz, DMSO-*d_6_*): *δ* = 1.27–1.36 (m, 3H, H_26_), 2.69–2.81 (m, 2H, H_14_), 3.87–3.94 (m, 1H, H_15_), 3.99–4.07 (m, 4H, H_16,25_), 6.77–6.88 (m, 2H, H_4_,_20_), 7.01–7.08 (m, 4H, H_2,6,8,12_), 7.17–7.24 (m, 3H, H_9,11,_), 7.31–7.65 (m, 4H, H_3,5,23,24_), 7.90 (s, 0.5H, H_18_), 8.05 (s, 0.5H, H_18_), 8.11 (s, 0.5H, NH), 8.52 (s, 0.5H, NH), 9.56–9.77 (m, 1H, OH), 11.30 (s, 0.5H, NH), 11.53 (s, 0.5H, NH); ^13^C NMR (101 MHz, DMSO-*d_6_*): *δ* = 15.0 (C_26_), 33.4 (C_15_), 35.2 (C_14_), 51.3 (C_16_), 64.2 (C_25_), 110.6, 111.0, 111.7, 116.7, 117.5, 120.0, 121.7, 122.6, 123.8, 125.8, 129.6, 132.0, 140.4, 143.9, 144.7, 147.5, 148.2, 149.3, 149.5, 150.4, 161.1, 169.1 (C_Ar,Ar′,Ar″_+C_18_), 172.2, 173.9 (C_13,17_). HRMS (ESI+): *m*/*z* calcd for C_26_H_27_N_4_O_4_ 459.2033 [M+H]^+^, found 459.2030.

*N′*-(naphthalen-2-ylmethylene)-5-oxo-1-(4-(phenylamino)phenyl)pyrrolidine-3-carbohydrazide (**11**).

Prepared from 1-naphthaldehyde. Yield 0.61 g (59%), dark green crystals, m.p. 201–202 °C; TLC: *R*_f_ = 0.2 (acetone/hexane = 1:1); IR (KBr) ν_max_ (cm^−1^): 1608, 1681 (C=O), 3030, 3389 (NH); ^1^H NMR (400 MHz, DMSO-*d_6_*): *δ* = 2.62–2.79 (m, 2H, H_14_), 3.97–4.14 (m, 3H, H_15,16_), 6.95 (t, 1H, *J* = 7.2 Hz, H_4_), 6.98–7.06 (m, 1H, H_2_), 7.39–7.46 (m, 1H, H_6_), 7.63–7.66 (m, 6H, H_3,5,8,9,11,12,_), 8.04 (d, 2H, H_22,25_), 8.10–8.14 (m, 3H, H_18,21,24_), 8.16 (s, 1H, NH), 9.12, 9.14 (2s, 2H, NH, H_27_), 9.41 (s, 2H, H_20,28_); ^13^C NMR (101 MHz, DMSO-*d_6_*): *δ* = 33.2 (C_15_), 35.3 (C_14_), 50.5 (C_16_), 107.3, 116.7, 117.2, 119.7, 121.7, 125.3, 126.0, 126.9, 128.1, 129.2, 129.5, 130.7, 131.0, 132.4, 133.9, 140.5, 162.4, 168.1 (C_Ar,Ar′,Ar″_+C_18_), 171.3, 173.8 (C_13,17_); HRMS (ESI+): *m*/*z* calcd for C_28_H_25_N_4_O_2_ 449.1978 [M+H]^+^, found 449.1968.

*N′*-((2-hydroxynaphthalen-1-yl)methylene)-5-oxo-1-(4-(phenylamino)phenyl)pyrrolidine-3-carbohydrazide (**12**) (E/Z isomeric mixture in DMSO-*d_6_* solution).

Prepared from 2-hydroxy-1-naphthaldehyde. Yield 0.61 g (57%), pink crystals, m.p. 229–230 °C; TLC: *R*_f_ = 0.33 (acetone/hexane = 1:1); IR (KBr) ν_max_ (cm^−1^): 1622, 1671 (C=O), 3031, 3189 (NH), 3357 (OH); ^1^H NMR (400 MHz, DMSO-*d_6_*): *δ* = 2.75–2.88 (m, 2H, H_14_), 3.42–3.46 (m, 1H, H_15_), 3.99–4.10 (m, 2H, H_16_), 6.80 (t, 1H, *J* = 7.2 Hz, H_4_), 7.05 (d, 2H, *J* = 7.2 Hz, H_2,6_), 7.11 (d, 2H, *J* = 7.4 Hz, H_3,5_), 7.19–7.24 (m, 3H, H_8,12,21_), 7.39 (t, 1H, *J* = 7.4 Hz, H_24_), 7.51–7.55 (m, 2H, H_9,11_), 7.59 (t, 1H, *J* = 7.4 Hz, H_25_), 7.84–7.92 (m, 2H, H_22,26_), 8.16 (s, 1H, NH), 8.28, 8.30 (2s, 0.8H, H_28_), 8.69, 8.71 (2s, 0.2H, H_28_), 8.95 (s, 0.2H, H_18_), 9.23 (s, 0.8H, H_18_), 10.90 (s, 0.2H, NH), 11.57 (s, 0.2H, OH), 11.99 (s, 0.8H, NH), 12.50 (s, 0.8H, OH); ^13^C NMR (101 MHz, DMSO-*d_6_*): *δ* = 33.1 (C_15_), 34.8, 35.5 (C_14_), 50.7 (C_16_), 108.5, 110.2, 116.2, 116.3, 117.2, 118.8, 119.4, 121.0, 121.1, 123.6, 127.8, 127.9, 129.0, 129.2, 131.5, 131.8, 132.8, 139.9, 143.7, 146.1, 156.9, 157.9, 168.4 (C_Ar,Ar′,Ar″_+C_18_), 171.1, 172.9 (C_13,17_). HRMS (ESI+): *m*/*z* calcd for C_28_H_25_N_4_O_3_ 465.1927 [M+H]^+^, found 465.1927.

### 3.2. Biological Activity

#### 3.2.1. Cell Culture

The human malignant melanoma cell line IGR39, human triple-negative breast cancer cell line MDA-MB-231, and human pancreatic carcinoma cell line Panc-1 were obtained from the American Type Culture Collection (ATCC, Manassas, VA, USA). Human foreskin fibroblasts (HFs) CRL-4001 were originally obtained from ATCC and kindly provided by Prof. Helder Santos (University of Helsinki, Helsinki, Finland). All cell lines were cultured in Dulbecco’s modified Eagle’s GlutaMAX medium (Gibco (Carlsbad, CA, USA)) supplemented with 10,000 U/mL penicillin, 10 mg/mL streptomycin (Gibco, Waltham, MA, USA), and 10% fetal bovine serum (Gibco). Cell cultures were cultured at 37 °C in a humidified atmosphere containing 5% CO_2_. They were used until passage 20.

#### 3.2.2. Cytotoxicity Assay

The effect of compounds on cell viability was established using the 3-(4,5-dimethylthiazol-2-yl)-2,5-diphenyltetrazolium bromide (MTT; Sigma-Aldrich Co., St Louis, MO, USA) assay, as described elsewhere [69]. Briefly, the cells were seeded in triplicate in 96-well TC-treated flat bottom plates (IGR39, MDA-MB-231, and Panc-1 cells: 4 × 10^3^ cells/well; HFs: 5 × 10^3^ cells/well). The cells were treated with 100 μM of the tested compounds after 24 h of incubation. After 72 h, the MTT reagent was added, and the formed formazan crystals were dissolved in DMSO (Sigma-Aldrich Co., St. Louis, MO, USA). The absorbance was determined with a multidetection microplate reader at 570 and 630 nm.

Using the same MTT procedure, the EC_50_ values of the most active hydrazones, namely, **4**, **6**, **8**, and **12**, and the clinically used drug sunitinib (SNT) (Sigma-Aldrich Co., St. Louis, MO, USA) were determined. The compounds were serially diluted to concentrations ranging from 50 µM to 1.56 µM and added to the cells. The Hill equation was used to calculate the EC_50_ value, or the concentration of a compound that results in a 50% reduction in the metabolic activity of cells.

#### 3.2.3. ‘Wound Healing’ Assay

The ‘wound healing’ assay was used to assess the ability of the most active compounds, **4**, **6**, **8**, and **12**, to inhibit cell migration, as published elsewhere [70]. Cancer cells were seeded at a density of 5 × 10^4^ cells/well in 24-well plates. After 48 h of incubation, a 100 µL pipette tip was used to make a scratch in the middle of each well. Following a single PBS wash, fresh medium containing the compounds at 10% and 50% of their EC_50_ were added to the cells. As a negative control, medium containing 0.1% DMSO was employed.

#### 3.2.4. Compound Activity Against Spheroids

The magnetic 3D bioprinting method was utilized to form cancer cell spheroids, as described elsewhere [41]. Briefly, the cells at 70% confluence were incubated with Nanoshuttle (n3D Biosciences, Inc., Houston, TX, USA) for 8 h. Then, the cells were trypsinized, centrifuged, and seeded into ultra-low attachment 96-well plates at a ratio of 1.5 × 10^3^ cancer cells and 1.5 × 10^3^ human fibroblasts/well. The plate was incubated for two days at 37 °C in a humidified atmosphere on a magnetic drive. Next, the medium with 20 µM of the selected compounds was added. Photos of spheroids were taken every two days using an Olympus IX73 inverted microscope (Olympus Corporation, Tokyo, Japan), and an analysis of the spheroid size was performed using ImageJ, version 1.53o (National Institutes of Health, Bethesda, MD, USA), and Microsoft Office Excel 2016 software (Microsoft Corporation, Redmond, WA, USA).

On the last day of the experiment, 20 µL of MTT reagent was added to each well. Following a 10-h incubation, the medium was aspirated, and the formazan crystals formed were dissolved in 100 µL of DMSO overnight. The absorbance was determined with a multidetector microplate reader Multiskan GO (Thermo Fisher Scientific Oy, Ratastie, Finland) at 570 and 630 nm.

#### 3.2.5. Statistical Analysis

All biological experiments were repeated at least three times, calculating the means and standard deviations. The data were processed using Microsoft Office Excel 2016 software (Microsoft Corporation, Redmond, WA, USA). The statistical analysis was performed using Student’s *t*-test. The level of significance was set as *p* < 0.05.

### 3.3. Molecular Docking

Molecular docking of the tested compounds **4**, **6**, **8**, and **12** as most potential protein kinase inhibitors was conducted using the *SchrödingerSuite 2024-1* software package [71]. The structures of target proteins EGFR (PDB: 1M17, 4HJO, 1XKK), VEGFR (PDB: 4AGD, 4ASD, 3EWH), HER2 (PDB: 3RCD, 3PPO), BRAF (PDB: 4RZV, 7MOU), MEK (PDB: 4U7Z, 7MOY), SCR (PDB: 1A07, 3F3V), and CDK5 (PDB: 1UNL) were retrieved from the Protein Data Bank (PDB) [72]. The target proteins were prepared using the Protein Preparation Wizard [73], involving removal of water molecules that do not form hydrogen bonds, completion of absent loops from the PDB file, and addition of hydrogen atoms absent in the structure of target protein. The next stage included the optimization of the protein structure at pH 7.0 using subprogram PROBKA and minimization of the protein structure using OPLS-2005. The structures of standard ligands vemurafenib, trametinib, and dasatinib that selectively bind to BRAF (1UWH), MEK (4U7Z), ACK-1 (5ZXB), and SCR (3F3V) were downloaded from the PDB database, where they were determined as reference ligands binding to the target kinases. The ligands’ preparation was conducted using LigPrep Wizard [74] by the determination of bonds’ orders and angles, coupled with minimization of the force field OPLS-2005. The ligand–receptor binding sites were generated using the Receptor Grid Generation tool in the Glide Maestro module. [75].

## 4. Conclusions

In conclusion, a series of novel hydrazone derivatives were synthesized from 5-oxo-1-(4-(phenylamino)phenyl)pyrrolidine-3-carbohydrazide in reactions with the corresponding aldehydes bearing one or more hydroxyl, alkoxy, and carboxylic groups on the benzene ring. An in vitro evaluation of the anticancer activity of the synthesized compounds was carried out and was supported by in silico molecular modeling results. Compounds **4**, **6**, **8**, and **12** were identified as the most promising anticancer agents among the synthesized hydrazone derivatives. 2-Hydroxybenzylidene derivative **8** and 2-hydroxynaphthalenylmethylene derivative **12** were more cytotoxic both in 2D and 3D assays, and 2,5-dimethoxybenzylidene derivative **4** and 2,4,6-trimethoxybenzylidene derivative **6** exhibited greater effects on inhibiting cell migration. Moreover, all compounds were less active against the Panc-1 cancer cell line in the cell monolayer, but the effect on spheroid cell viability in 3D cultures was quite similar among the tested cancer cell lines.

Molecular modeling results for the most active hydrazones **4**, **6**, **8**, and **12** have indicated that these compounds may act as multikinase inhibitors. *N′*-((2-hydroxynaphthalen-1-yl)methylene)-5-oxo-1-(4-(phenylamino)phenyl)pyrrolidine-3-carbohydrazide (**12**) showed high affinity values for the active sites of two types of protein kinases simultaneously, which indicates the potential of a dual mechanism of kinase inhibition for this molecule.

## Figures and Tables

**Table 1:** Ligand–protein docking score values for compounds **4**, **6**, **8**, and **12**.

Ligand	Non-Receptor Tyrosine Kinases				Serine Threonine Protein Kinases			
	ACK-1 (PDB: 5ZXB)		SCR (PDB: 3F3V)		BRAF (PDB: 1UWH)		MEK (PDB: 4U7Z)	
	Docking Score kcal/mol	Glide Emodel kcal/mol	Docking Score kcal/mol	Glide Emodel kcal/mol	Docking Score kcal/mol	Glide Emodel kcal/mol	Docking Score kcal/mol	Glide Emodel kcal/mol
**4**	−9.604	−85.377	−10.113	−99.691	−10.546	−103.191	−10.115	−104.676
**6**	−10.057	−98.268	−10.067	−98.824	−10.313	−105.035	−10.588	−124.007
**8**	−10.272	−88.632	−9.907	−101.847	−10.504	−90.466	−10.885	−171.810
**12**	−9.929	−96.430	−11.174	−113.405	−11.471	−101.653	−9.158	−145.819
Dasatinib	−9.412	−108.418	−9.758	−115.364	−10.428	−101.336	−8.052	−104.318
Trametinib	−7.171	−67.301	−5.636	−37.185	−10.256	−62.904	−9.545	−111.156
Vemurafenib	−8.322	−86.139	−7.201	−70.350	−11.313	−96.824	−7.275	−89.902

## Data Availability

The original contributions presented in this study are included in the article and Supplementary Materials. Further inquiries can be directed to the corresponding authors.

## Supplementary Materials

The following supporting information can be downloaded at https://www.mdpi.com/article/10.3390/ijms26073162/s1.
