# Supplementary material for: Novel 5-Oxopyrrolidine-3-carbohydrazides as Potent Protein Kinase Inhibitors: Synthesis, Anticancer Evaluation, and Molecular Modeling
Source: Int J Mol Sci. 2025 Mar 29;26(7):3162. doi: 10.3390/ijms26073162 (PMC11989890; doi:10.3390/ijms26073162)
Supplement: Supplementary file 1 [file ijms-26-03162-s001.zip › ijms-3530935-supplementary.pdf]

Supplementary Materials

Novel 5-Oxopyrrolidine-3-carbohydrazides as Potent Protein Kinase Inhibitors: Synthesis, Anticancer Evaluation and Molecular Modeling

Ingrida Tumosienė [1], Maryna Stasevych [2], Viktor Zvarych [3], Ilona Jonuškienė [1], Kristina Kantminienė [4,*] and Vilma Petrikaitė [5,6,*]

Department of Organic Chemistry, Kaunas University of Technology, Radvilėnų pl. 19, 50254 Kaunas, Lithuania; ingrida.tumosiene@ktu.lt (I.T.), ilona.jonuskiene@ktu.lt (I.J.)

2 Department of Technology of Biologically Active Substances, Pharmacy, and Biotechnology, Lviv Polytechnic National University, S. Bandera Str. 12, 79013 Lviv, Ukraine; maryna.v.stasevych@gmail.com

3 Department of Automated Control Systems, Lviv Polytechnic National University, S. Bandera Str. 12, 79013 Lviv, Ukraine; viktor.i.zvarych@lpnu.ua

4 Department of Physical and Inorganic Chemistry, Kaunas University of Technology, Radvilėnų pl. 19, 50254 Kaunas, Lithuania; kristina.kantminiene@ktu.lt

5 Laboratory of Drug Targets Histopathology, Institute of Cardiology, Lithuanian University of Health Sciences, Sukilėlių pr. 13, 50162 Kaunas, Lithuania; vilma.petrikaite@lsmuni.lt

6 Institute of Biotechnology, Life Sciences Center, Vilnius University, Saulėtekio al. 7, 10257 Vilnius, Lithuania

* Correspondence: kristina.kantminiene@ktu.lt (K.K.); vilma.petrikaite@lsmuni.lt (V.P.)

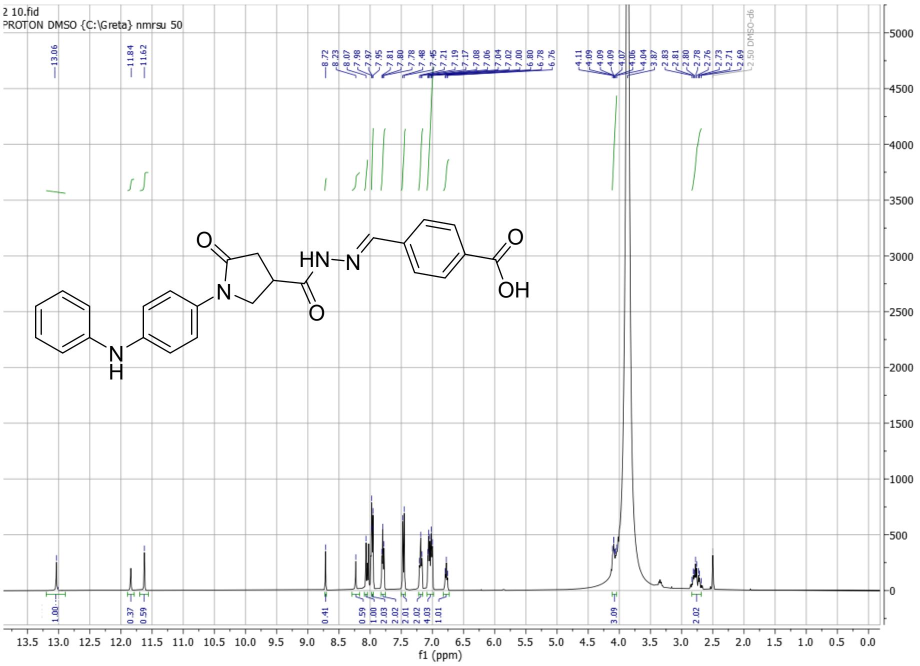

Figure S1. ^{1}H NMR (400 MHz, DMSO-d_6) spectrum of **2**

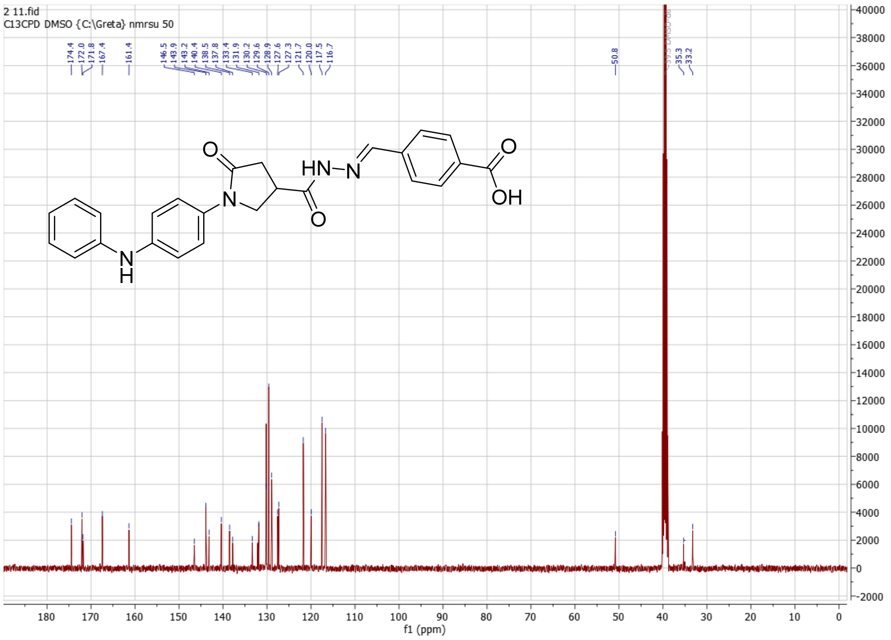

Figure S2. ^{13}C NMR (101 MHz, DMSO-d_6) spectrum of **2**

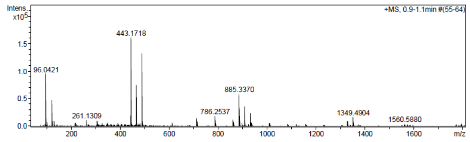

Meas. m/z	#	Formula	Score	m/z	err [ppm]	Mean err [ppm]	mSigma	rdb	e⁻ Conf	N-Rule
443.1718	1	C 25 H 23 N 4 O 4	100.00	443.1714	-0.9	-0.7	2.0	16.5	even	ok
	2	C 24 H 27 O 8	38.16	443.1700	-4.0	-3.6	12.0	11.5	even	ok
	3	C 26 H 19 N 8	59.92	443.1727	2.1	2.3	16.0	21.5	even	ok
	4	C 20 H 24 Cl N 8 O 2	0.08	443.1705	-2.9	-2.8	162.0	12.5	even	ok
	5	C 24 H 28 Cl N 2 O 4	0.05	443.1732	3.2	3.3	166.3	11.5	even	ok
	6	C 19 H 29 Cl 2 N 6 O 2	0.00	443.1724	1.3	1.4	284.1	7.5	even	ok
465.1538	1	C 26 H 25 O 8	100.00	465.1544	1.3	1.5	4.5	14.5	even	ok
	2	C 23 H 17 N 10 O 2	89.90	465.1530	-1.6	-1.5	5.6	20.5	even	ok
	3	C 27 H 21 N 4 O 4	39.77	465.1557	4.1	4.4	9.7	19.5	even	ok
	4	C 22 H 22 Cl N 8 O 2	0.08	465.1549	2.3	2.4	165.1	15.5	even	ok
	5	C 21 H 26 Cl N 4 O 6	0.11	465.1535	-0.6	-0.4	166.4	10.5	even	ok
	6	C 20 H 30 Cl O 10	0.04	465.1522	-3.4	-3.3	168.8	5.5	even	ok
	7	C 20 H 31 Cl 2 N 2 O 6	0.00	465.1554	3.4	3.5	288.5	5.5	even	ok
488.2298	1	C 27 H 30 N 5 O 4	100.00	488.2292	-1.2	-0.9	3.8	15.5	even	ok
	2	C 28 H 26 N 9	83.92	488.2306	1.5	1.8	9.7	20.5	even	ok
	3	C 26 H 34 N O 8	35.80	488.2279	-4.0	-3.6	14.4	10.5	even	ok
	4	C 22 H 31 Cl N 9 O 2	0.30	488.2284	-3.0	-3.6	141.7	11.5	even	ok
	5	C 26 H 35 Cl N 3 O 4	0.26	488.2311	2.5	2.0	146.2	10.5	even	ok
885.3370	1	C 50 H 45 N 8 O 8	61.74	885.3355	-1.7	-1.5	6.7	32.5	even	ok
	2	C 51 H 41 N 12 O 4	100.00	885.3368	-0.2	-0.1	19.5	37.5	even	ok
	3	C 54 H 49 N 2 O 10	60.48	885.3382	1.3	1.5	20.1	31.5	even	ok

Figure S3. HRMS spectrum of **2**

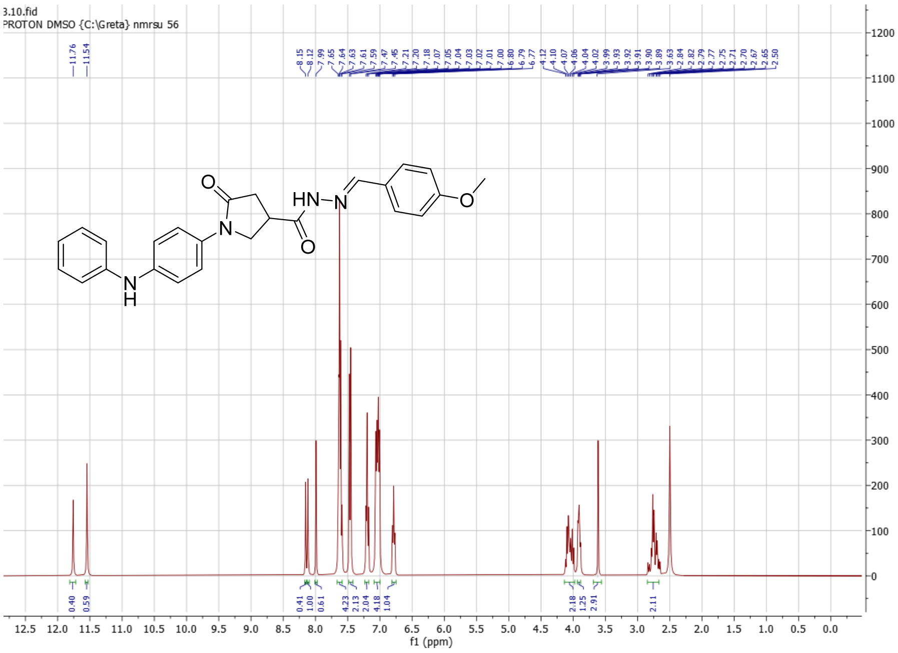

Figure S4. ^{1}H NMR (400 MHz, DMSO-*d*6) spectrum of **3**

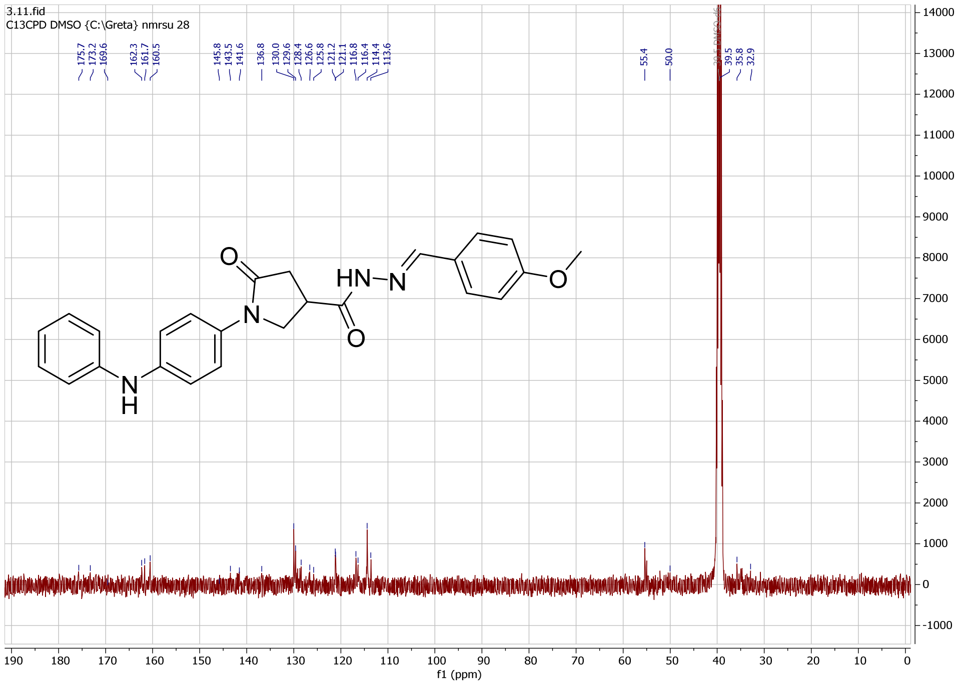

Figure S5. ^{13}C NMR (101 MHz, DMSO-*d*6) spectrum of **3**

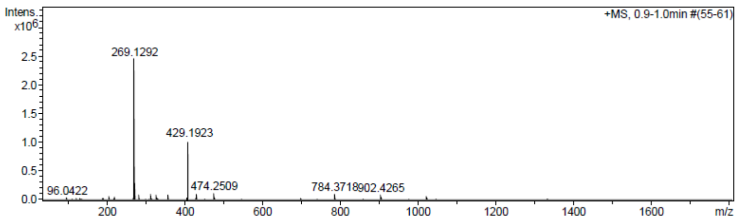

Meas. m/z	#	Formula	Score	m/z	err [ppm]	Mean err [ppm]	mSigma	rdb	e⁻ Conf	N-Rule
269.1292	1	C 16 H 17 N 2 O 2	100.00	269.1285	-2.6	-2.4	28.4	9.5	even	ok
429.1923	1	C 25 H 25 N 4 O 3	100.00	429.1921	-0.5	-0.3	11.7	15.5	even	ok
	2	C 24 H 29 O 7	42.39	429.1908	-3.6	-3.4	17.9	10.5	even	ok
474.2509	1	C 27 H 32 N 5 O 3	100.00	474.2500	-1.9	-1.4	26.2	14.5	even	ok

Figure S6. HRMS spectrum of **3**

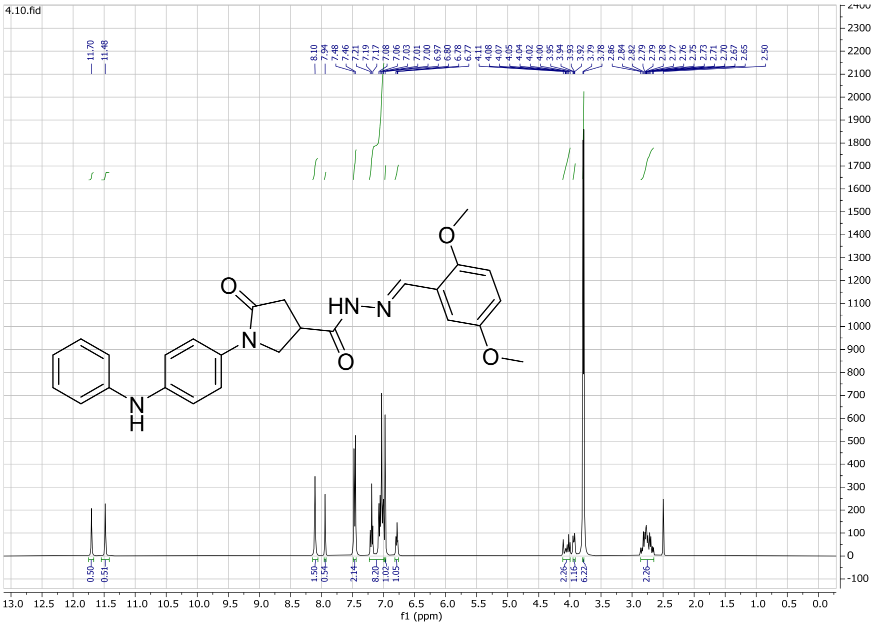

Figure S7. ^{1}H NMR (400 MHz, DMSO-d_6) spectrum of **4**

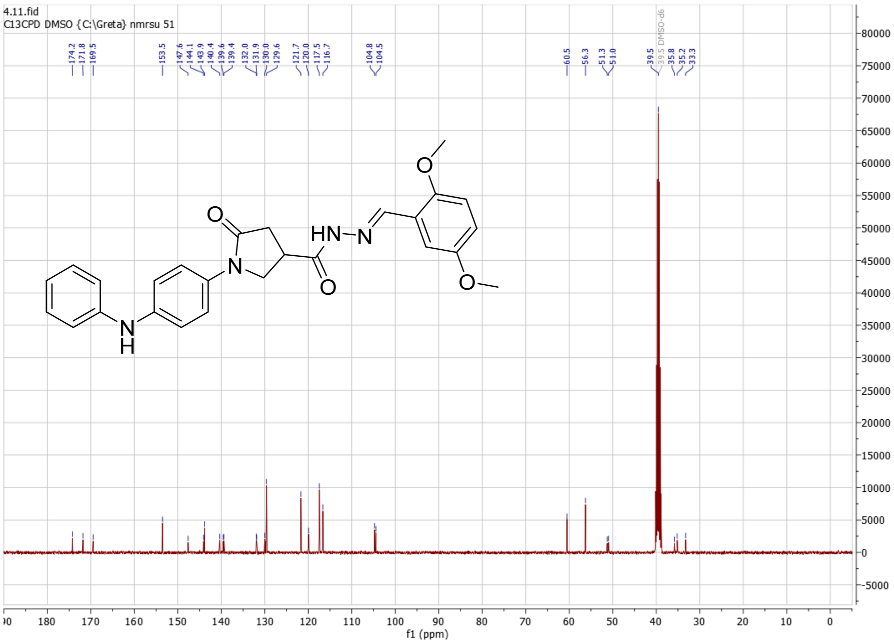

Figure S8. ^{13}C NMR (101 MHz, DMSO-d_6) spectrum of **4**

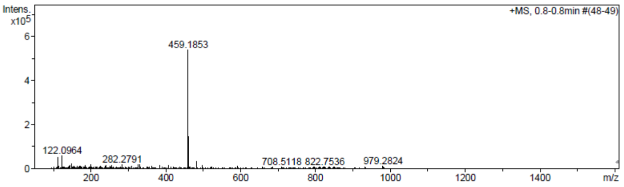

Meas. m/z	#	Formula	Score	m/z	err [ppm]	Mean err [ppm]	mSigma	rdb	e⁻ Conf	N-Rule
459.1853	1	C 21 H 31 O 11	99.63	459.1861	1.6	2.7	16.7	6.5	even	ok
	2	C 19 H 19 N 14 O	100.00	459.1861	1.6	2.4	16.8	17.5	even	ok
	3	C 20 H 25 N 7 O 6	98.87	459.1861	1.6	2.4	17.2	12.0	odd	ok
	4	C 24 H 25 N 7 O S	49.49	459.1836	-3.8	-4.1	19.7	16.0	odd	ok
	5	C 25 H 31 O 6 S	48.96	459.1836	-3.8	-4.0	20.4	10.5	even	ok
	6	C 18 H 23 N 10 O 5	91.63	459.1847	-1.3	-0.5	23.7	12.5	even	ok
	7	C 19 H 29 N 3 O 10	90.80	459.1847	-1.3	-0.5	24.3	7.0	odd	ok

Figure S9. HRMS spectrum of **4**

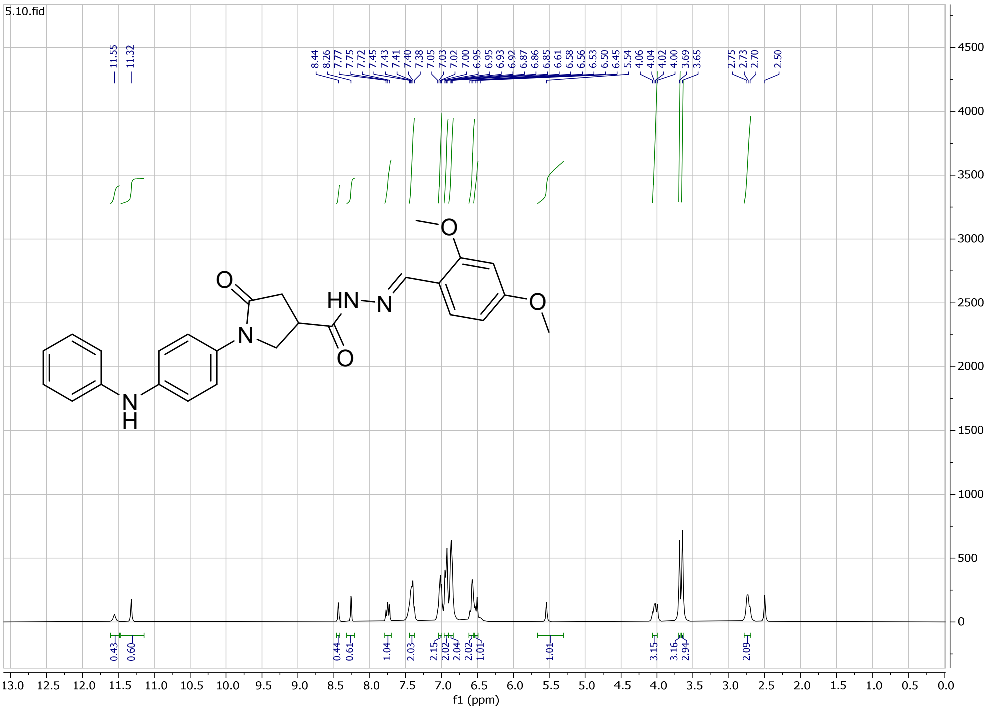

Figure S10. ^{1}H NMR (400 MHz, DMSO-d_6) spectrum of **5**

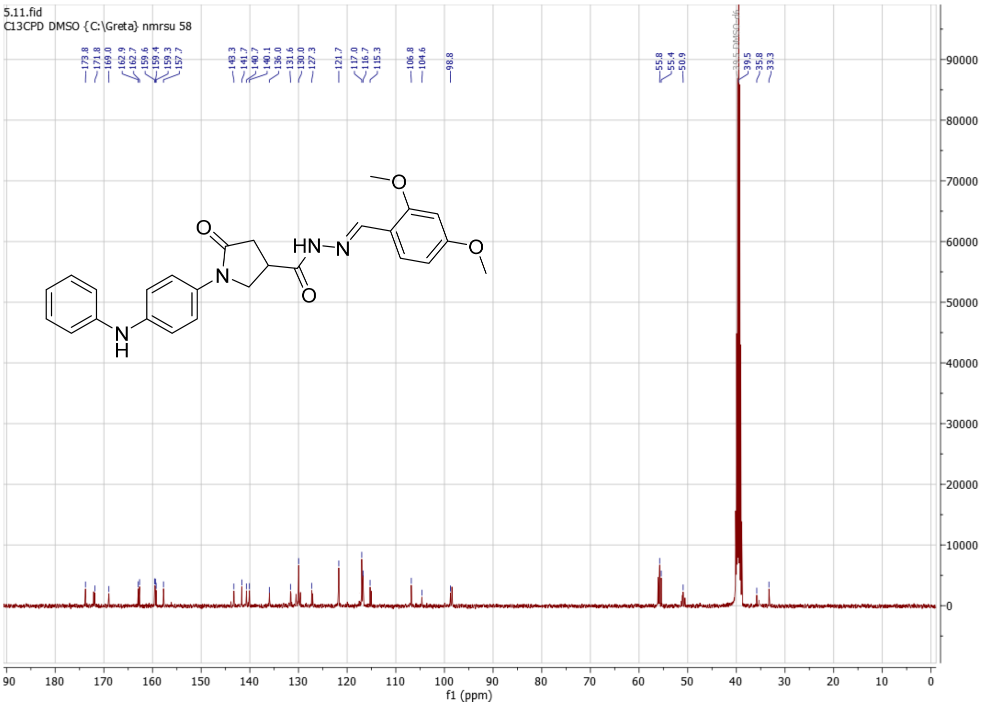

Figure S11. ^{13}C NMR (101 MHz, DMSO-d_6) spectrum of **5**

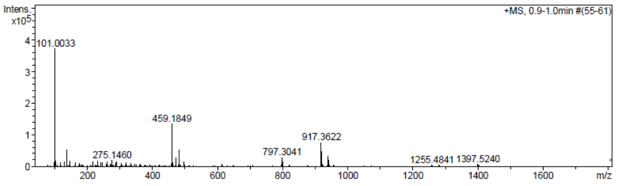

Meas. m/z	#	Formula	Score	m/z	err [ppm]	Mean err [ppm]	mSigma	rdb	e⁻ Conf	N-Rule
459.1849	1	C 26 H 27 N 4 O 2 S	100.00	459.1849	-0.1	-0.5	7.3	15.5	even	ok
	2	C 25 H 31 O 6 S	42.51	459.1836	-3.0	-3.4	15.9	10.5	even	ok
	3	C 34 H 23 N 2	43.08	459.1856	1.4	2.6	33.7	24.5	even	ok
	4	C 21 H 31 O 11	26.30	459.1861	2.5	3.7	41.6	6.5	even	ok

Figure S12. HRMS spectrum of **5**

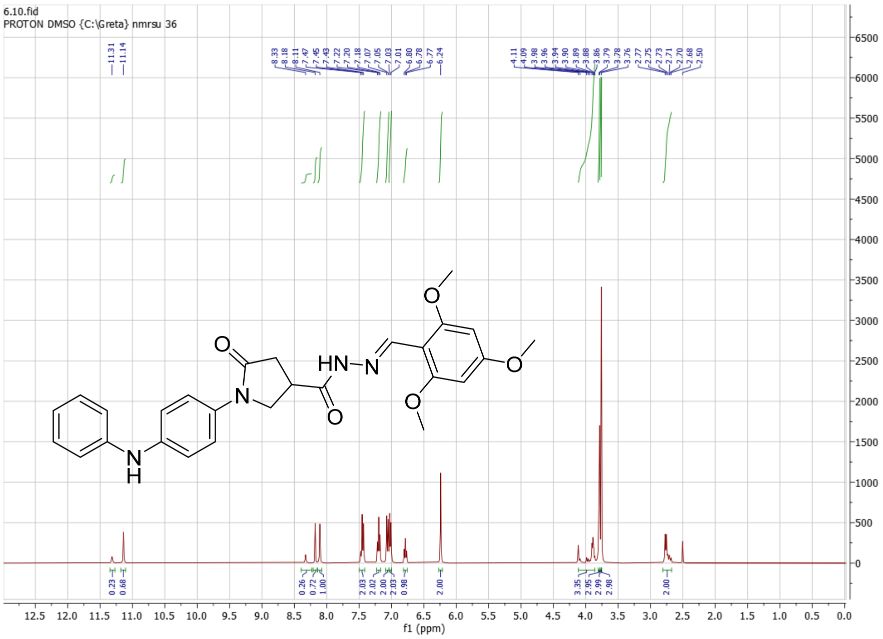

Figure S13. ^{1}H NMR (400 MHz, DMSO-d_6) spectrum of **6**

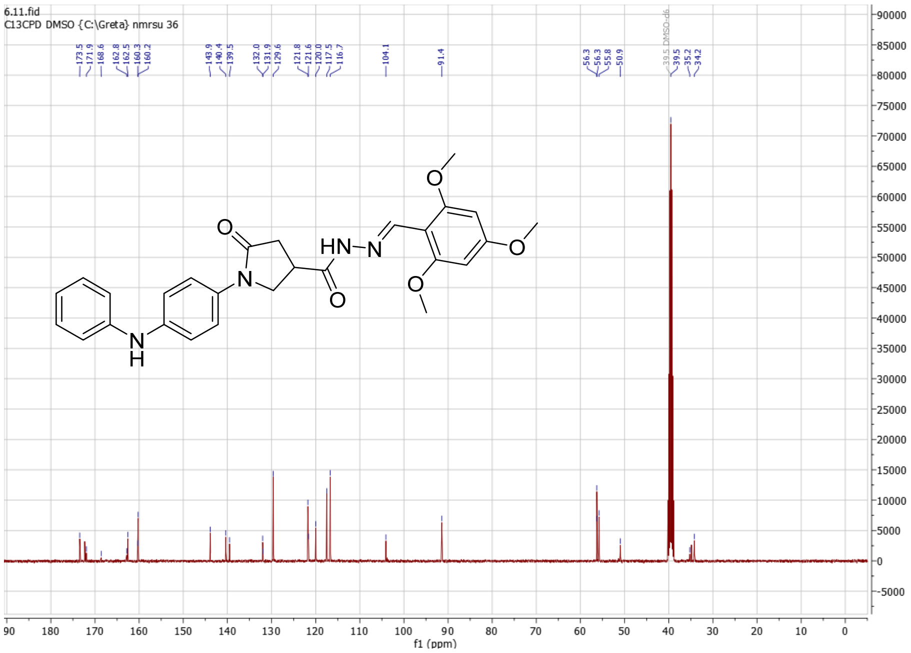

Figure S14. ^{13}C NMR (400 MHz, DMSO-d_6) spectrum of **6**

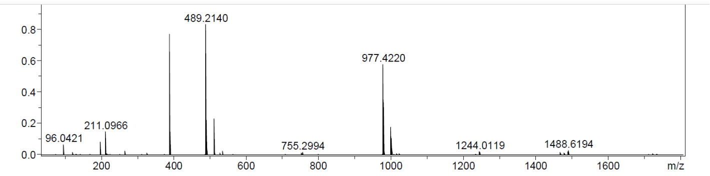

Meas. m/z	#	Formula	Score	m/z	err [ppm]	Mean err [ppm]	mSigma	rdb	e⁻ Conf	N-Rule
389.1711	1	C 20 H 25 N 2 O 6	100.00	389.1707	-1.0	-0.7	18.3	9.5	even	ok
	2	C 17 H 17 N 12	43.29	389.1694	-4.5	-4.3	20.7	15.5	even	ok
	3	C 21 H 21 N 6 O 2	54.20	389.1721	2.4	2.7	32.8	14.5	even	ok
	4	C 16 H 22 Cl N 10	0.30	389.1712	0.2	0.4	155.3	10.5	even	ok
	5	C 15 H 26 Cl N 6 O 4	0.16	389.1699	-3.2	-3.1	156.2	5.5	even	ok
	6	C 19 H 30 Cl O 6	0.10	389.1725	3.7	3.9	160.5	4.5	even	ok
	7	C 15 H 27 Cl 2 N 8	0.00	389.1730	4.9	5.1	276.6	5.5	even	ok
	8	C 14 H 31 Cl 2 N 4 O 4	0.00	389.1717	1.5	1.7	277.9	0.5	even	ok
489.2140	1	C 27 H 29 N 4 O 5	100.00	489.2132	-1.6	-1.3	21.3	15.5	even	ok
	2	C 28 H 25 N 8 O	82.93	489.2146	1.2	1.4	33.5	20.5	even	ok
	3	C 22 H 30 Cl N 8 O 3	0.30	489.2124	-3.3	-3.8	146.3	11.5	even	ok
	4	C 26 H 34 Cl N 2 O 5	0.29	489.2151	2.2	1.8	152.7	10.5	even	ok
	5	C 21 H 35 Cl 2 N 6 O 3	0.00	489.2142	0.4	0.0	271.4	6.5	even	ok
	6	C 20 H 39 Cl 2 N 2 O 7	0.00	489.2129	-2.3	-2.7	271.5	1.5	even	ok
[illegible]	[illegible]	[illegible]	[illegible]	[illegible]	[illegible]	[illegible]	[illegible]	[illegible]		[illegible]

Figure S15. HRMS spectrum of **6**

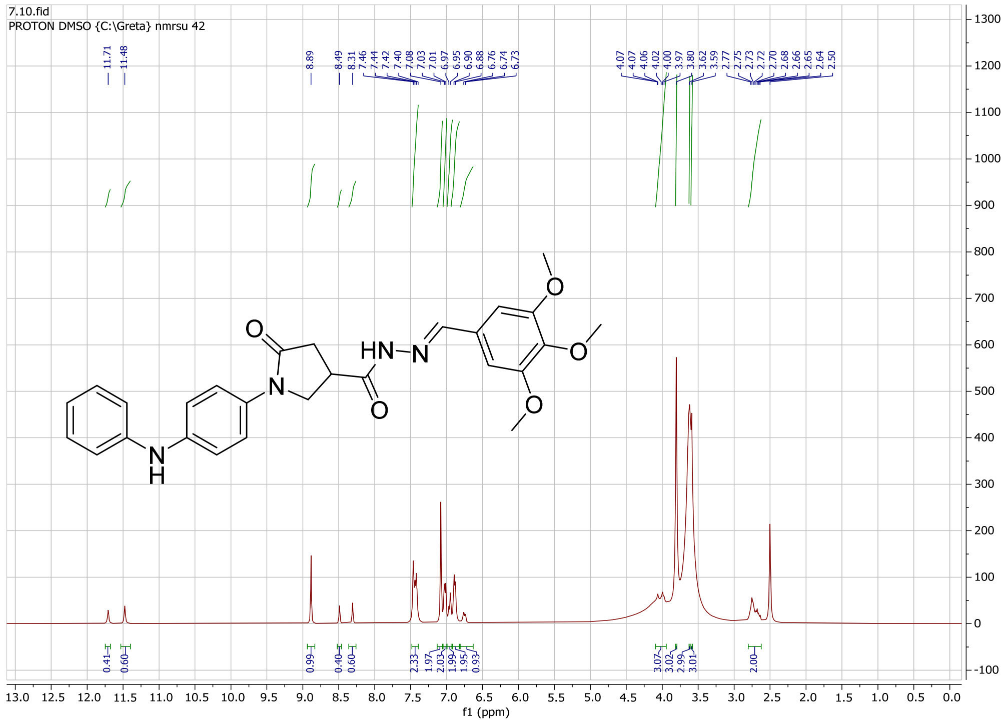

Figure S16. ^{1}H NMR (400 MHz, DMSO-d_6) spectrum of **7**

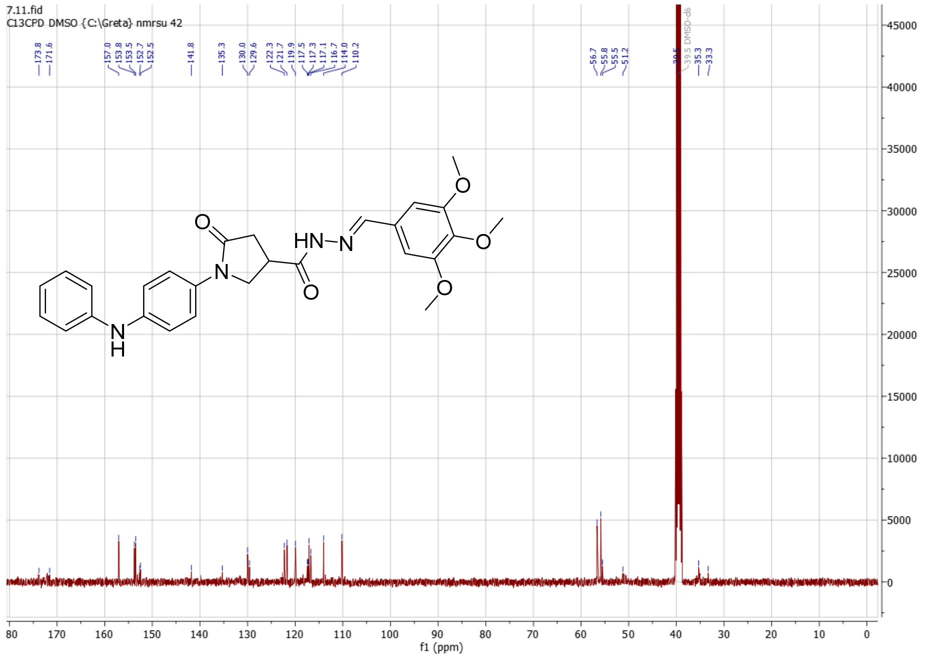

Figure S17. ^{13}C NMR (101 MHz, DMSO-d_6) spectrum of **7**

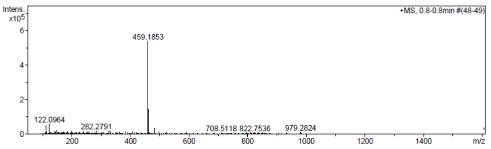

Meas. m/z	#	Formula	Score	m/z	err [ppm]	Mean err [ppm]	mSigma	rdb	e⁻ Conf	N-Rule
459.1853	1	C 21 H 31 O 11	99.63	459.1861	1.6	2.7	16.7	6.5	even	ok
	2	C 19 H 19 N 14 O	100.00	459.1861	1.6	2.4	16.8	17.5	even	ok
	3	C 20 H 25 N 7 O 6	98.87	459.1861	1.6	2.4	17.2	12.0	odd	ok
	4	C 24 H 25 N 7 O S	49.49	459.1836	-3.8	-4.1	19.7	16.0	odd	ok
	5	C 25 H 31 O 6 S	48.96	459.1836	-3.8	-4.0	20.4	10.5	even	ok
	6	C 18 H 23 N 10 O 5	91.63	459.1847	-1.3	-0.5	23.7	12.5	even	ok
	7	C 19 H 29 N 3 O 10	90.80	459.1847	-1.3	-0.5	24.3	7.0	odd	ok
	8	C 26 H 27 N 4 O 2 S	94.83	459.1849	-0.9	-1.1	26.1	15.5	even	ok
	9	C 28 H 29 N O 3 S	64.00	459.1863	2.0	1.8	32.0	15.0	odd	ok
	10	C 34 H 23 N 2	46.63	459.1856	0.5	1.4	55.1	24.5	even	ok

Figure S18. HRMS spectrum of **7**

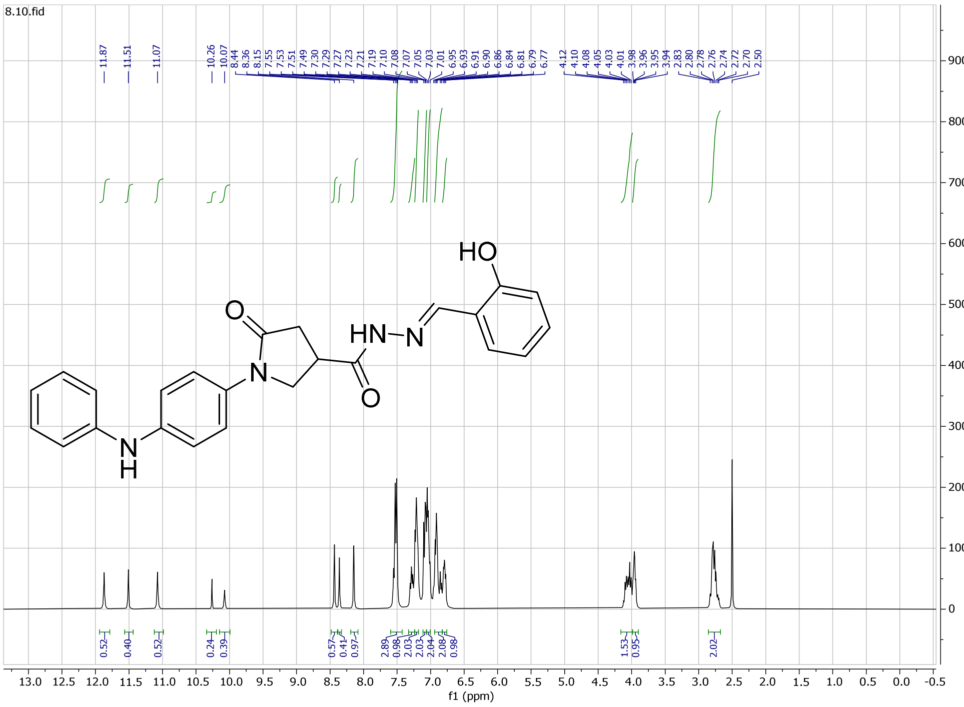

Figure S19. ^{1}H NMR (400 MHz, DMSO-d_6) spectrum of **8**

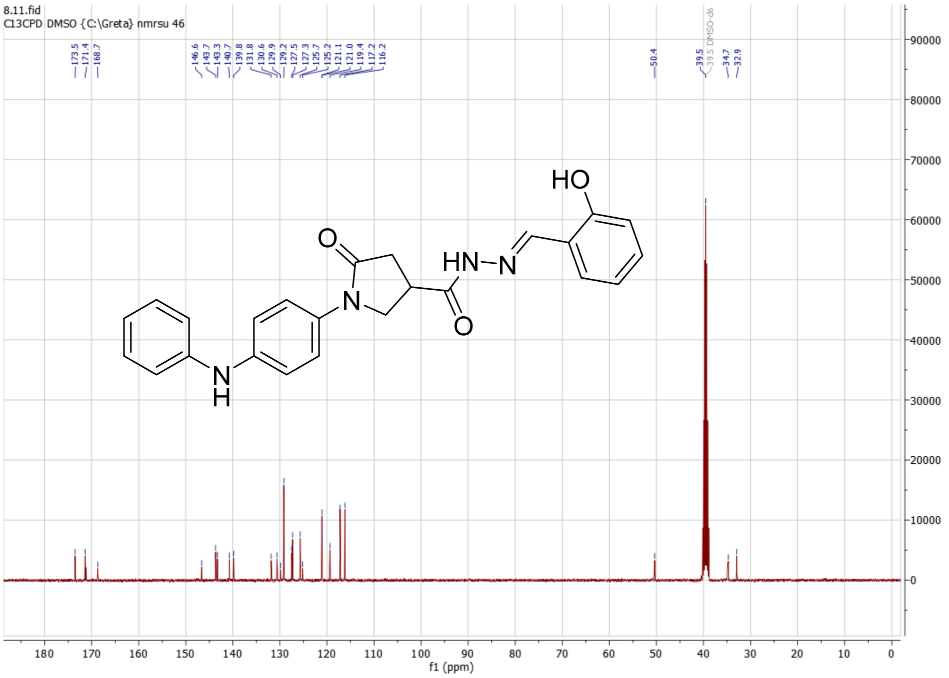

Figure S20. ^{13}C NMR (101 MHz, DMSO-d_6) spectrum of **8**

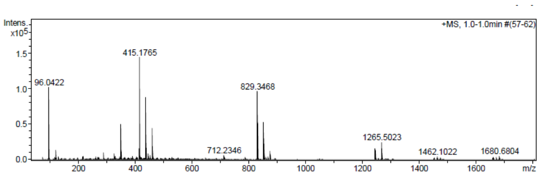

Meas. m/z	#	Formula	Score	m/z	err [ppm]	Mean err [ppm]	mSigma	rdb	e⁻ Conf	N-Rule
415.1765	1	C 24 H 23 N 4 O 3	100.00	415.1765	-0.1	0.0	5.8	15.5	even	ok
	2	C 23 H 27 O 7	47.79	415.1751	-3.4	-3.1	8.1	10.5	even	ok
437.1586	1	C 25 H 25 O 7	89.38	437.1595	2.0	2.2	2.9	13.5	even	ok
	2	C 22 H 17 N 10 O	100.00	437.1581	-1.1	-0.9	7.1	19.5	even	ok
	3	C 21 H 21 N 6 O 5	41.46	437.1568	-4.2	-4.0	11.2	14.5	even	ok
460.2346	1	C 26 H 30 N 5 O 3	100.00	460.2343	-0.5	-0.4	3.4	14.5	even	ok
	2	C 25 H 34 N O 7	43.74	460.2330	-3.4	-3.3	8.3	9.5	even	ok
829.3468	1	C 48 H 45 N 8 O 6	80.22	829.3457	-1.3	-1.0	4.3	30.5	even	ok
	2	C 52 H 49 N 2 O 8	47.91	829.3483	1.9	2.3	16.3	29.5	even	ok
	3	C 49 H 41 N 12 O 2	100.00	829.3470	0.3	0.6	16.8	35.5	even	ok
851.3284	1	C 50 H 43 N 8 O 6	47.23	851.3300	1.9	2.1	7.4	33.5	even	ok
	2	C 49 H 47 N 4 O 10	99.66	851.3287	0.3	0.5	7.5	28.5	even	ok
	3	C 47 H 35 N 18	100.00	851.3287	0.3	0.4	7.6	39.5	even	ok
	4	C 46 H 39 N 14 O 4	65.03	851.3273	-1.3	-1.1	7.8	34.5	even	ok
	5	C 48 H 51 O 14	55.60	851.3273	-1.3	-1.0	16.3	23.5	even	ok

Figure S21. HRMS spectrum of **8**

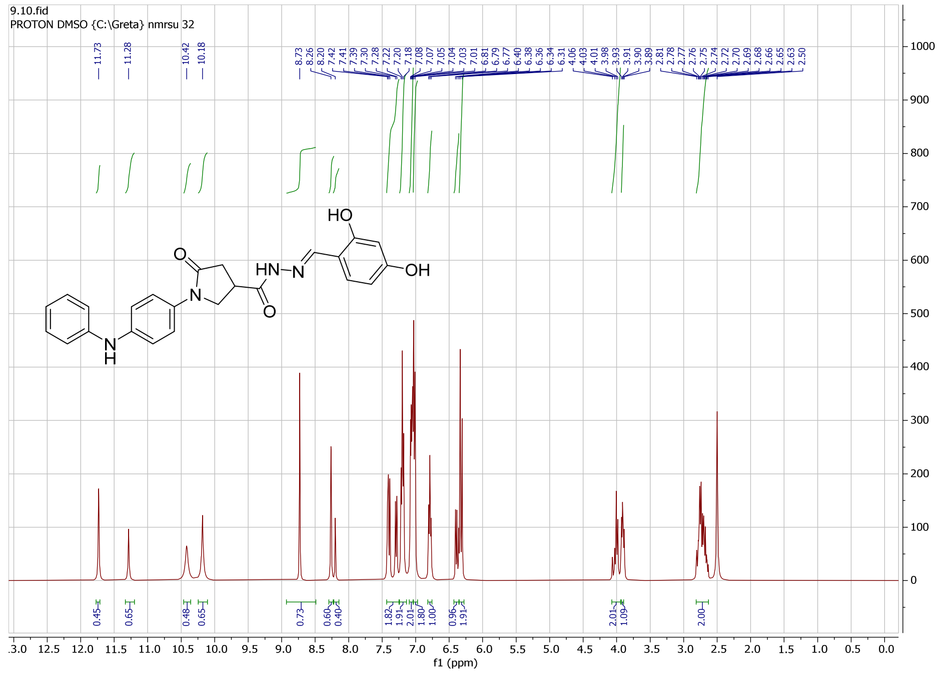

Figure S22. ^{1}H NMR (400 MHz, DMSO-d_6) spectrum of **9**

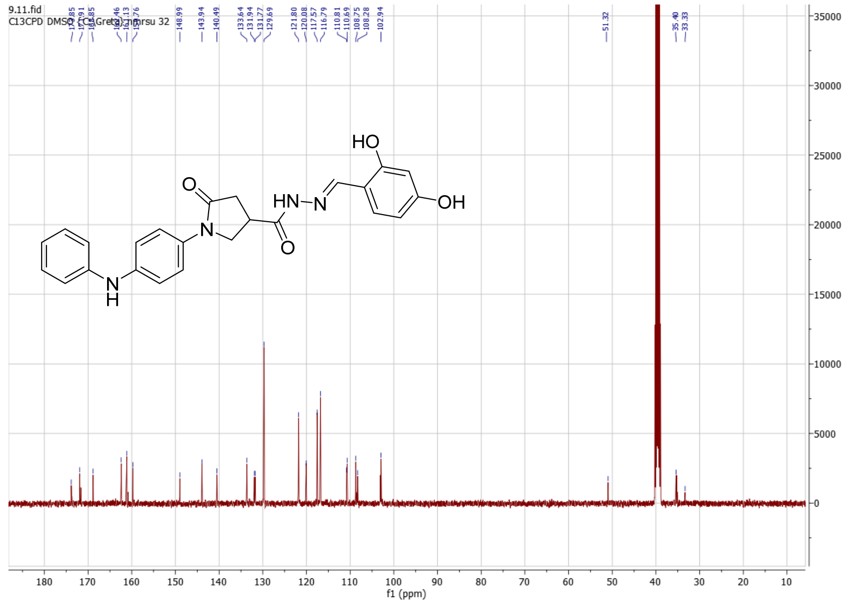

Figure S23. ^{13}C NMR (101 MHz, DMSO-d_6) spectrum of **9**

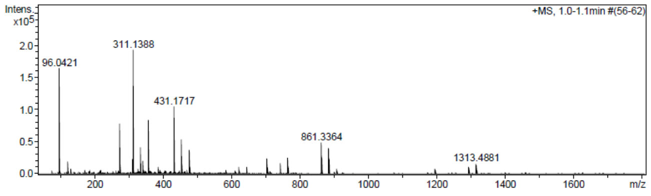

Meas. m/z	#	Formula	Score	m/z	err [ppm]	Mean err [ppm]	mSigma	rdb	e⁻ Conf	N-Rule
311.1388	1	C 18 H 19 N 2 O 3	100.00	311.1390	0.7	0.9	9.3	10.5	even	ok
356.1970	1	C 20 H 26 N 3 O 3	100.00	356.1969	-0.5	-0.2	3.3	9.5	even	ok
431.1717	1	C 24 H 23 N 4 O 4	100.00	431.1714	-0.7	-0.5	5.0	15.5	even	ok
	2	C 25 H 19 N 8	63.43	431.1727	2.4	2.5	9.8	20.5	even	ok
	3	C 23 H 27 O 8	37.10	431.1700	-3.8	-3.6	17.2	10.5	even	ok
453.1535	1	C 22 H 17 N 10 O 2	100.00	453.1530	-0.9	-0.8	10.6	19.5	even	ok
	2	C 25 H 25 O 8	74.97	453.1544	2.0	2.3	12.5	13.5	even	ok
	3	C 21 H 21 N 6 O 6	33.46	453.1517	-3.9	-3.7	25.3	14.5	even	ok
861.3364	1	C 48 H 45 N 8 O 8	100.00	861.3355	-1.1	-0.9	12.3	30.5	even	ok
	2	C 52 H 49 N 2 O 10	44.88	861.3382	2.1	2.3	24.8	29.5	even	ok
	3	C 49 H 41 N 12 O 4	98.44	861.3368	0.5	0.7	25.2	35.5	even	ok

Figure S24. HRMS spectrum of **9**

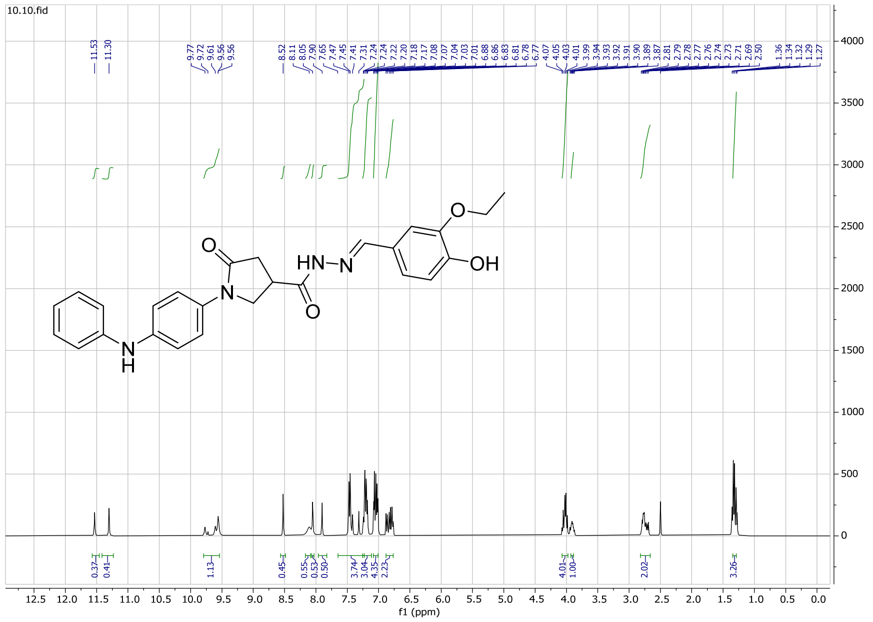

Figure S25. ^{1}H NMR (400 MHz, DMSO-d_6) spectrum of **10**

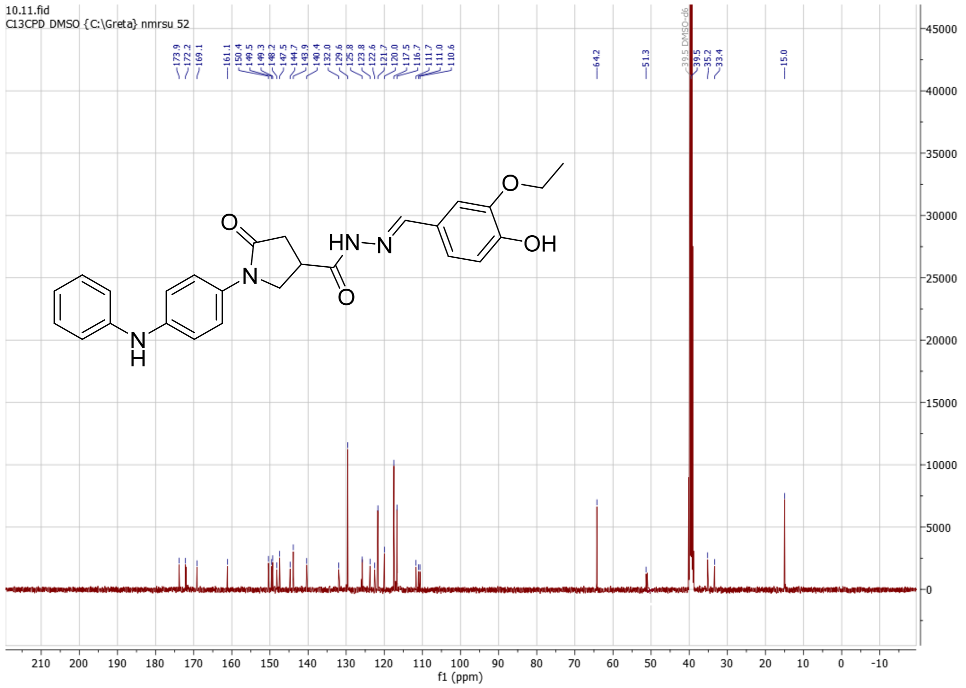

Figure S26. ^{13}C NMR (101 MHz, DMSO-d_6) spectrum of **10**

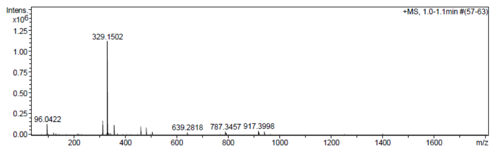

Meas. m/z	#	Formula	Score	m/z	err [ppm]	Mean err [ppm]	mSigma	rdb	e⁻ Conf	N-Rule
329.1502	1	C 18 H 21 N 2 O 4	100.00	329.1496	-1.7	-1.5	31.0	9.5	even	ok
	2	C 19 H 17 N 6	61.21	329.1509	2.3	2.6	45.5	14.5	even	ok
459.2030	1	C 26 H 27 N 4 O 4	100.00	459.2027	-0.8	-0.5	6.7	15.5	even	ok
	2	C 27 H 23 N 8	70.24	459.2040	2.1	2.4	8.5	20.5	even	ok
	3	C 25 H 31 O 8	38.45	459.2013	-3.7	-3.4	16.4	10.5	even	ok
481.1850	1	C 27 H 29 O 8	100.00	481.1857	1.4	1.6	5.8	13.5	even	ok
	2	C 24 H 21 N 10 O 2	98.36	481.1843	-1.4	-1.3	6.5	19.5	even	ok

Figure S27. HRMS spectrum of **10**

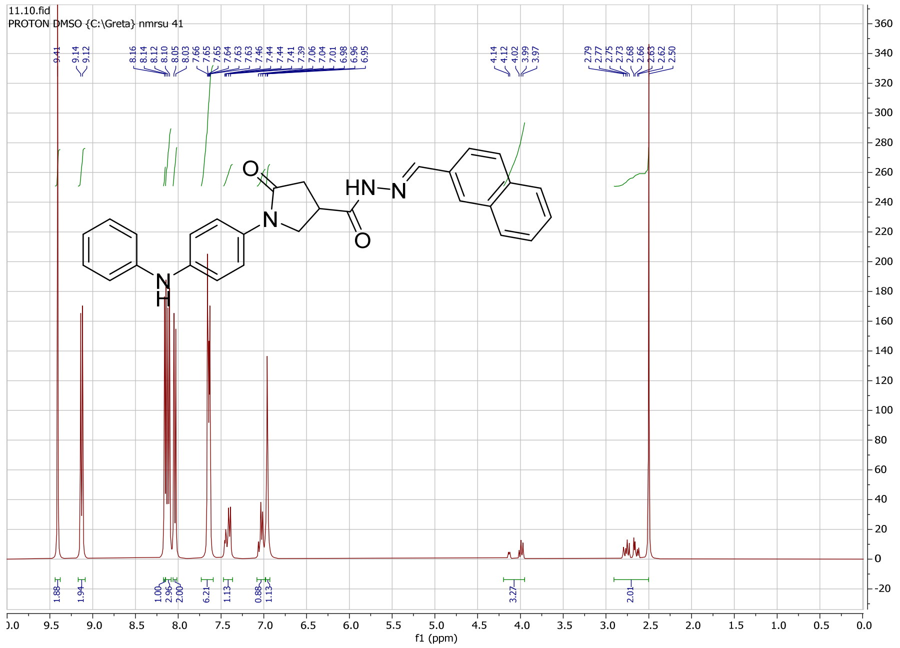

Figure S28. ^{1}H NMR (400 MHz, DMSO-d_6) spectrum of **11**

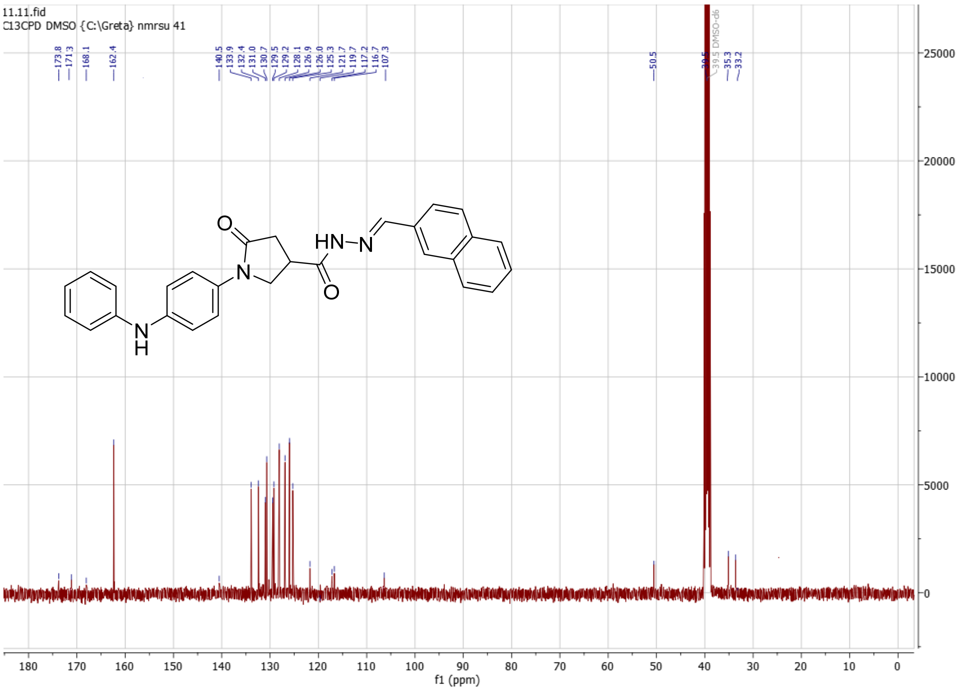

Figure S29. ^{13}C NMR (101 MHz, DMSO-d_6) spectrum of **11**

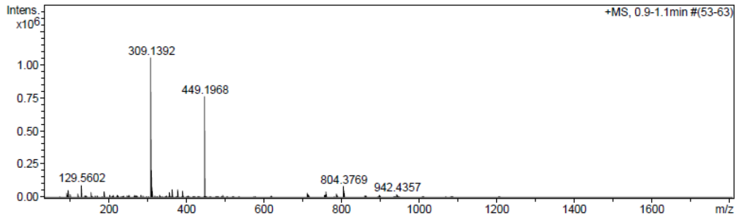

Meas. m/z	#	Formula	Score	m/z	err [ppm]	Mean err [ppm]	mSigma	rdb	e⁻ Conf	N-Rule
309.1392	1	C 22 H 17 N 2	100.00	309.1386	-1.9	-0.9	35.9	15.5	even	ok
363.1499	1	C 25 H 19 N 2 O	100.00	363.1492	-2.1	-2.0	22.1	17.5	even	ok
377.1654	1	C 26 H 21 N 2 O	100.00	377.1648	-1.4	-1.4	9.4	17.5	even	ok
391.1900	1	C 26 H 23 N 4	51.15	391.1917	4.3	4.9	9.0	17.5	even	ok
	2	C 25 H 27 O 4	100.00	391.1904	0.9	1.5	14.2	12.5	even	ok
449.1968	1	C 28 H 25 N 4 O 2	100.00	449.1972	0.9	2.3	50.1	18.5	even	ok
	2	C 27 H 29 O 6	61.46	449.1959	-2.0	-0.6	57.6	13.5	even	ok
712.2349	1	C 47 H 30 N 5 O 3	100.00	712.2343	-0.8	4.1	237.6	35.5	even	ok
	2	C 46 H 34 N O 7	29.41	712.2330	-2.7	2.3	241.6	30.5	even	ok

Figure S30. HRMS spectrum of **11**

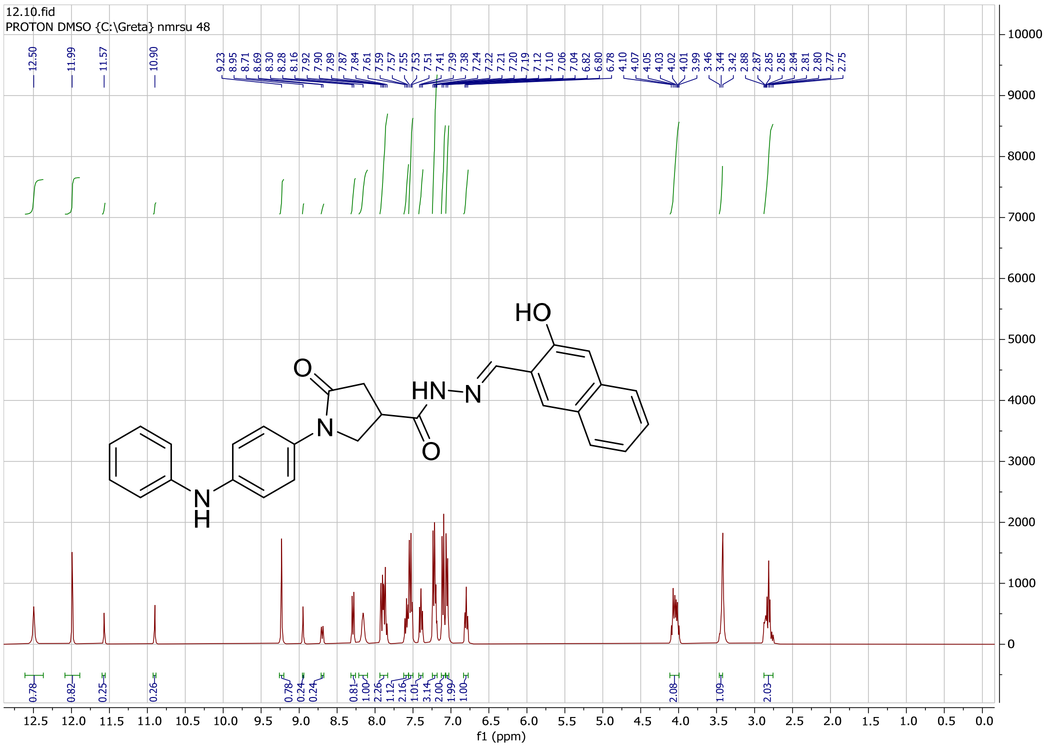

Figure S31. ^{1}H NMR (400 MHz, DMSO-*d*$_6$) spectrum of **12**

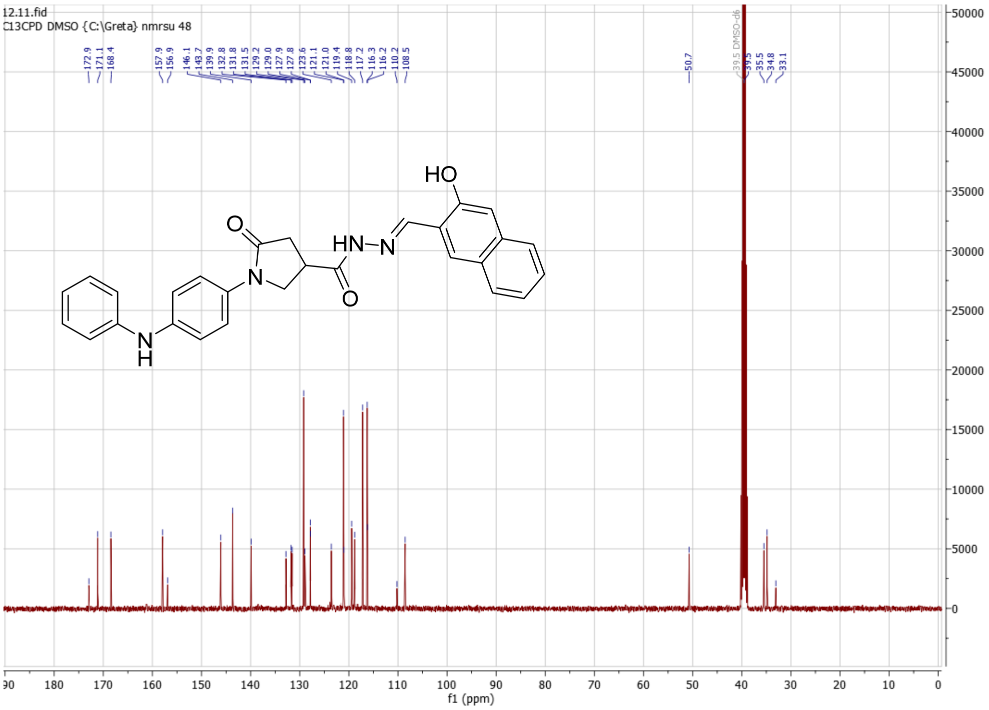

Figure S32. ^{13}C NMR (101 MHz, DMSO-*d*$_6$) spectrum of **12**

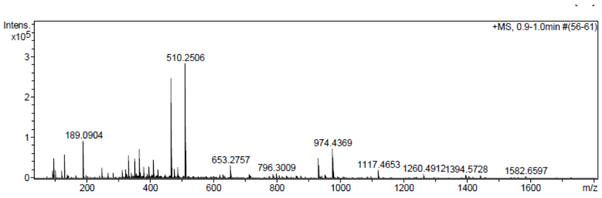

Meas. m/z	#	Formula	Score	m/z	err [ppm]	Mean err [ppm]	mSigma	rdb	e¯ Conf	N-Rule
465.1927	1	C 27 H 29 O 7	46.67	465.1908	-4.0	-3.7	4.9	13.5	even	ok
	2	C 28 H 25 N 4 O 3	100.00	465.1921	-1.2	-0.9	7.9	18.5	even	ok
510.2506	1	C 30 H 32 N 5 O 3	100.00	510.2500	-1.3	-1.0	16.8	17.5	even	ok
974.4369	1	C 62 H 60 N 3 O 8	100.00	974.4375	0.6	0.7	8.2	34.5	even	ok
	2	C 63 H 56 N 7 O 4	40.20	974.4388	2.0	2.0	12.4	39.5	even	ok

Figure S33. HRMS spectrum of **12**
